# Formation of Linear Amplicons with Inverted Duplications in *Leishmania* Requires the MRE11 Nuclease

**DOI:** 10.1371/journal.pgen.1004805

**Published:** 2014-12-04

**Authors:** Marie-Claude N. Laffitte, Marie-Michelle Genois, Angana Mukherjee, Danielle Légaré, Jean-Yves Masson, Marc Ouellette

**Affiliations:** 1 Centre de Recherche en Infectiologie du CHU de Québec, Quebec City, Québec, Canada; 2 Genome Stability Laboratory, CHU de Quebec Research Center, HDQ Pavillon, Oncology Axis, Quebec City, Québec, Canada; 3 Department of Molecular Biology, Medical Biochemistry and Pathology, Laval University, Quebec City, Québec, Canada; The University of North Carolina at Chapel Hill, United States of America

## Abstract

Extrachromosomal DNA amplification is frequent in the protozoan parasite *Leishmania* selected for drug resistance. The extrachromosomal amplified DNA is either circular or linear, and is formed at the level of direct or inverted homologous repeated sequences that abound in the *Leishmania* genome. The RAD51 recombinase plays an important role in circular amplicons formation, but the mechanism by which linear amplicons are formed is unknown. We hypothesized that the *Leishmania infantum* DNA repair protein MRE11 is required for linear amplicons following rearrangements at the level of inverted repeats. The purified LiMRE11 protein showed both DNA binding and exonuclease activities. Inactivation of the *LiMRE11* gene led to parasites with enhanced sensitivity to DNA damaging agents. The MRE11^−/−^ parasites had a reduced capacity to form linear amplicons after drug selection, and the reintroduction of an *MRE11* allele led to parasites regaining their capacity to generate linear amplicons, but only when MRE11 had an active nuclease activity. These results highlight a novel MRE11-dependent pathway used by *Leishmania* to amplify portions of its genome to respond to a changing environment.

## Author Summary

Extrachromosomal DNA amplification is frequent in the human protozoan parasite *Leishmania* when challenged with drug or other stressful conditions. DNA amplicons, either circular or linear, are formed by recombination between direct or inverted repeats spread throughout the genome of the parasite. The recombinase RAD51 is involved in the formation of circular amplicons, but the mechanism by which linear amplicons are formed is still unknown in this parasite. Studies in other organisms have provided some evidence that a DNA break is required for linear amplifications, and that the DNA repair protein MRE11 can be involved in this process. In this work, we present our biochemical, cellular and molecular characterization of the *Leishmania infantum MRE11* orthologue and provide evidence that this nuclease is involved in the formation of linear amplicons in *Leishmania*. Our results highlight a novel MRE11-dependent pathway used by *Leishmania* to amplify portions of its genome to respond to a changing environment.

## Introduction

**Figure 1:**
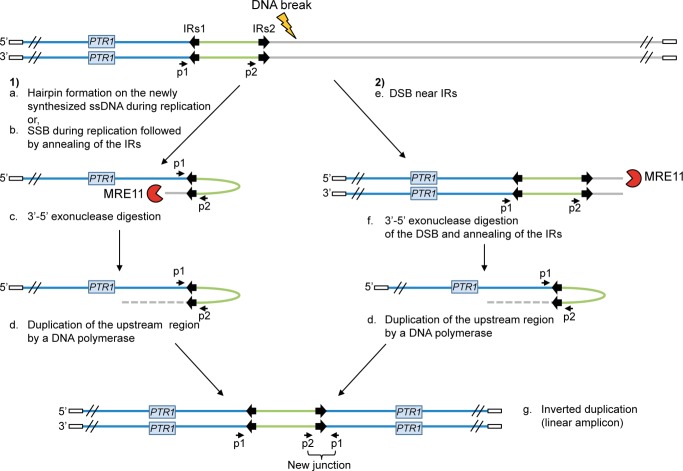
Potential mechanisms for the formation of extrachromosomal linear amplicons. (**1**) Single-strand hairpin formation (a) or single-strand break (SSB) (near the IRs) during replication followed by annealing of the IRs (b), 3′-5′ exonuclease digestion of the exposed end (c) and DNA synthesis of the upstream region (d) and of the second strand to form an inverted duplication (g). (**2**) Double-strand break (DSB) near the IRs (e) followed by 3′-5′ exonuclease digestion at the DNA break of one strand (f), annealing of the IRs to form an inverted duplication (d) and synthesis of the second strand to generate linear amplicon (g). The new junction formed during annealing of the IRs can be detected by PCR using specific primers. IRs, inverted repeated sequences; ss, single-strand; SSB, single-strand break; DSB, double-strand break; p1–p2, primer pair used to detect the new junction. White rectangle: telomeric sequences.

The protozoan parasite *Leishmania* is responsible for a group of diseases named leishmaniasis, affecting approximately 12 million people worldwide. No vaccine is currently available against *Leishmania* and treatments mainly rely on chemotherapy [1], [2]. Pentavalent antimony is the main anti-leishmanial drug although treatment failure due to resistance has been reported in most endemic regions [3]–[7]. Locus amplification is a frequent resistance mechanism allowing the parasite to modulate gene copy number and increased gene expression. Indeed, the parasite *Leishmania* is an early diverging eukaryotic parasite with no control of gene expression at the level of transcription initiation [8]–[10] and amplification of DNA loci is one strategy to increase the expression of resistance genes. Resistance genes can be amplified as part of extrachromosomal circular DNAs (circular amplicons) or as inverted duplications (linear amplicons) under drug pressure [11]–[16]. Gene rearrangements leading to locus amplification always occur at the level of either homologous direct or inverted repeated (IRs) sequences leading respectively to circular or linear extrachromosomal amplification [14], [15], [17]–[19]. A model for the generation of linear amplicons is shown in Figure 1.

A recent bioinformatics screen revealed that repeated sequences are widely distributed in the *Leishmania* genome, which is continuously being rearranged at the level of those repeated sequences. This process is adaptive as the copy number of advantageous extrachromosomal circular or linear elements increases upon selective pressure [19]. The whole genome of *Leishmania* is thus stochastically rearranged at the level of repeated sequences and the selection of parasite subpopulations with changes in the copy number of specific loci is used as one strategy to respond to drug pressure.

Circular or linear amplification has been observed when parasites were selected against a wide variety of drugs including the mainstay antimony [15], [20] but one drug that has proven highly useful in deciphering gene amplification mechanisms in *Leishmania* is the model antifolate drug methotrexate (MTX). Two loci are frequently amplified after MTX selection, one encoding the dihydrofolate reductase-thymidylate synthase (*DHFR-TS*) gene, the target of MTX, usually as part of circular elements [14], [21]–[23] the second encoding the pteridine reductase 1 (*PTR1*) gene, which is less sensitive to MTX but can reduce folates when DHFR-TS is blocked [24], [25]. The *PTR1* gene is amplified as part of either circular amplicons [22], [26], [27] or linear amplicons [13], [14], [19], [28], [29].

We have recently provided mechanistic insights into the formation of circular amplicons mediated by homologous recombination between direct repeated sequences catalyzed by the RAD51 recombinase [19]. However rearrangements at IRs leading to linear amplicons were not RAD51-dependent. Studies in other organisms have provided some evidence that a DNA break is required for palindromic amplifications formed by annealing of IRs [30]–[33] and that an exonuclease activity must be recruited to perform DNA end resection after chromosomal breakage in order to allow annealing of IRs [34]–[36] (see also Figure 1). Based on these observations, we hypothesized the involvement of the nuclease MRE11 (Meiotic REcombination 11) in the generation of linear amplicons in *Leishmania* (Figure 1). MRE11 interacts with RAD50 and NBS1 to form the MRN complex [37], [38]. Indeed, the nuclease MRE11 is a sensor of DNA double-strand breaks in cells and is important for the DNA double-strand break repair pathway [39], [40] by homologous recombination (HR) or non-homologous end joining (NHEJ) [41]. *Leishmania infantum* encodes a putative MRE11 with conserved endo- and exonuclease domains as well as DNA-binding domains [41]. In this manuscript, we present our biochemical, cellular and molecular characterization of the *L. infantum* MRE11 orthologue and provide evidence that this nuclease is involved in the formation of linear amplicons in the parasite *Leishmania*.

## Results

### Purification and biochemical characterization of LiMRE11

**Figure 2:**
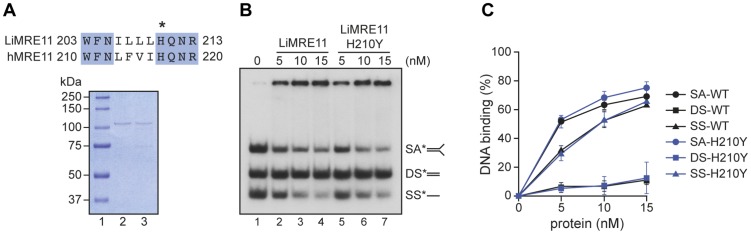
Purification and DNA binding of the *L. infantum* MRE11 protein. (**A**) Alignment of *L. infantum* and human MRE11 proteins showing the conserved catalytic residue (H) that has been mutated in LiMRE11 (H210Y) to generate the LiMRE11^H210Y^ mutated version and purification of LiMRE11^WT^ and LiMRE11^H210Y^ followed by SDS–PAGE separation. Purified proteins (150 ng) were loaded on an 8% SDS-PAGE, run then stained with Coomassie blue (GE Healthcare). Lane 1: molecular weight markers (Bio-Rad Laboratories); lane 2: purified LiMRE11^WT^; lane 3: purified LiMRE11^H210Y^. (**B**) LiMRE11^WT^ and mutant H210Y can bind various DNA structures. Competition electrophoretic mobility shift assays were performed with LiMRE11^WT^ (lanes 2–4) and LiMRE11^H210Y^ (lanes 5–7) and 25 nM of ssDNA (SS), dsDNA (DS) and splayed arm (SA) substrates with increasing concentration of the proteins (0, 5, 10, 15 nM). (**C**) Quantification of the DNA binding signals of panel **B**.

Since the critical catalytic residues of MRE11 are conserved in *Leishmania* [41] and the replacement of the histidine (H) at position 217 by a tyrosine (Y) is known to abolish the nuclease activity of the human MRE11 but not its nucleic acid binding property [42], we scrutinized the amino acid alignment between the human and *Leishmania* sequences and found the equivalent of human H217 at position 210 of LiMRE11 (Figure 2A, upper panel and Figure S1). We therefore produced a LiMRE11 mutated at the corresponding amino acid (LiMRE11^H210Y^) and used a two-step affinity purification procedure to purify LiMRE11^WT^ and LiMRE11^H210Y^ as described in Material and Methods (Figure 2A, lower panel).

We used the electrophoretic mobility shift assay to study DNA interactions with MRE11 proteins (Figure 2B). We observed that the splayed arm (SA) and single-strand (SS) DNA structures were shifted in the presence the wild-type and mutated MRE11 protein in a dose-dependent manner while neither version of the protein were able to shift the double-strand (DS) structure in this competitive assay. The binding was quantitated and at 15 nM of either protein, 65% binding was observed with either SS and SA DNA structures while we observed only 10% binding for DS DNA (Figure 2C).

**Figure 3:**
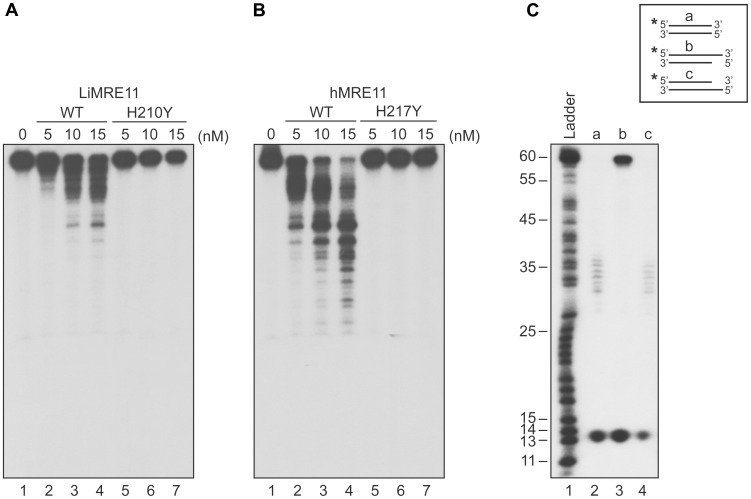
Exonuclease assays of MRE11 proteins. The *Leishmania* (**A**) and human (**B**) WT MRE11 proteins can perform DNA resection (lanes 1, 2, 3, 4) on dsDNA. Resection activity of the *Leishmania* MRE11^H210Y^ (**A**) and human MRE11^H217Y^ (**B**) (lanes 5, 6, 7). (**C**) Substrate specificity of LiMRE11^WT^ for resection activity. Two hundred nM of blunt dsDNAs (lane 2), 3′ overhang (lane 3) and 5′overhang (lane 4) were incubated with 25 nM of purified LiMRE11^WT^ protein and run on an 8% acrylamide/urea gel, followed by autoradiography.

We next tested whether purified LiMRE11^WT^ and LiMRE11^H210Y^ displayed exonuclease activity (Figure 3A), in comparison with human MRE11^WT^ and hMRE11^H217Y^ proteins (Figure 3B). Our findings suggest that LiMRE11^WT^ is enzymatically active and can perform exonucleolytic degradation with a 3′ to 5′ polarity but it is less effective than the human MRE11^WT^ protein in cleaving DNA into smaller fragments (Figure 3A–B, lanes 1–4). As expected, LiMRE11^H210Y^ was unable to perform DNA resection (Figure 3A, lanes 5–7), similar to its human mutated counterpart (Figure 3B, lanes 5–7). The substrate specificity was also monitored by using 25 nM of LiMRE11^WT^ protein with DS DNA, either blunt or with 3′ or 5′ overhang DNA structures (Figure 3C). The same extensive degradation was observed with DS DNA and 5′-overhang ends (Figure 3C, lane 2 and 4) while the protein was blocked by 3′-overhang extremities (Figure 3C, lane 3). LiMRE11^WT^ also exhibits endonuclease activity, as shown by the 13 bp band found at the bottom of the gel.

### LiMRE11-GFP is recruited at DNA damage loci in human MRE11-deficient cells (ATLD)

**Figure 4:**
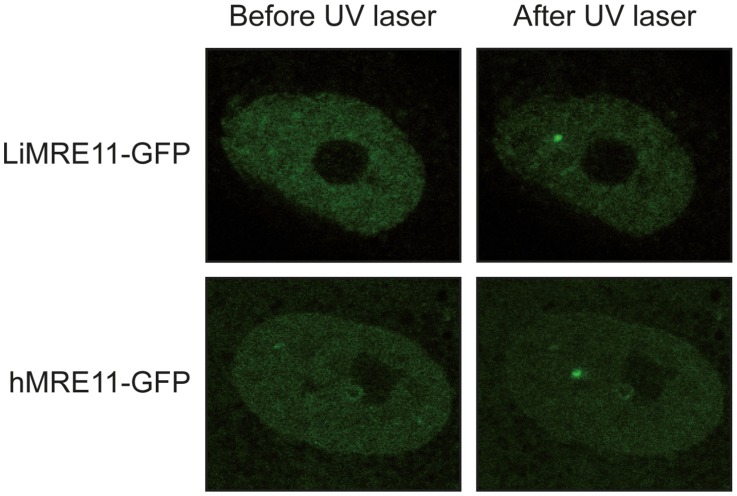
Fluorescence recovery after photobleaching analysis after DNA damage induction in UV-irradiated cells. LiMRE11-GFP is recruited to DNA damage sites in human MRE11-deficient cells (ATLD), as the human MRE11-GFP.

We generated a LiMRE11-GFP fusion construct that was transfected in *L. infantum* cells. However, we could never achieve a high copy number of the plasmid (overexpression of MRE11 can be toxic to the cell, see below) and fluorescence levels were too low for analysis. We then turned to a heterologous system to study LiMRE11 in vivo. DNA constructs encoding the fusion protein LiMRE11^WT^-GFP and the human counterpart hMRE11^WT^-GFP were transfected in human ATLD cells, which are deficient for hMRE11 [43]. After laser-induced DNA damage in these cells, we detected a localized fluorescent foci representative of the recruitment of LiMRE11^WT^-GFP in micro-irradiated nuclear regions (Figure 4, upper panels), similar to what was observed for the human MRE11^WT^-GFP fusion protein (Figure 4, bottom panels). Among 24 ATLD cells micro-irradiated, we observed a recruitment of LiMRE11 to DNA damages sites in 75% of the cases, while the human homolog was recruited in 100% of the cells. No recruitment was observed for the control GFP alone. These observations confirmed the ability of the *Leishmania* MRE11 protein to be recruited at DNA damage sites, in a heterologous cellular model. Altogether, these results show that LiMRE11 display similar localization properties as the human enzyme.

### Inactivation of the *Leishmania infantum MRE11* gene

**Figure 5:**
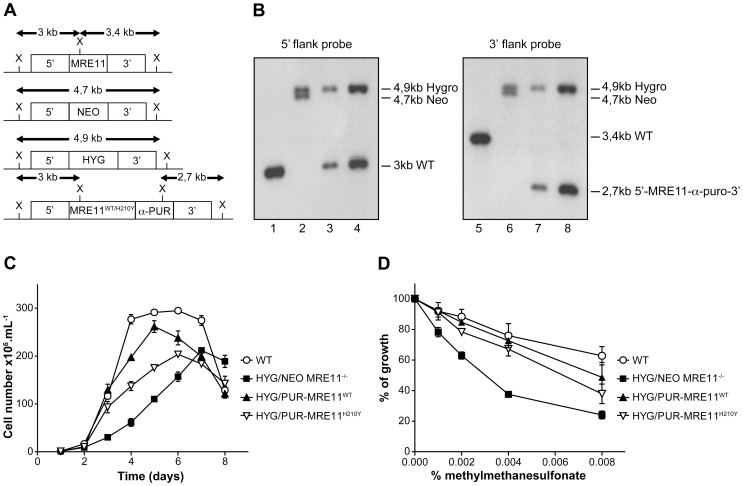
*MRE11* gene inactivation in *L. infantum* and phenotypic analysis. (**A**) Schematic representation of the *MRE11* locus in *L. infantum* before and after integration of the inactivation cassettes neomycin phosphotransferase (5′-*NEO-3′*) and hygromycin phosphotransferase B (5′-*HYG-3′*) generating the double knockout strain *HYG/NEO MRE11^−/−^*. A revertant was obtained by the integration of the re-expressing *MRE11* ^WT^ or *MRE11* ^H210Y^ puromycin cassettes (5′-*MRE11* ^WT^-α-*PUR-3′* and *5′-MRE11* ^H210Y^-*α-PUR-3′*) to replace the *NEO* allele, given respectively strains *HYG/PUR-MRE11* ^WT^ and *HYG/PUR-MRE11* ^H210Y^. X, XhoI restriction sites. (**B**) Southern blot analysis with genomic DNAs digested with XhoI from the *L. infantum* WT strain (lanes 1 and 5) and recombinant clones of the double knockout *HYG/NEO MRE11^−/−^* (lanes 2 and 6), *HYG/PUR-MRE11* ^WT^ (lanes 3 and 7) and *HYG/PUR-MRE11* ^H210Y^ parasites (lanes 4 and 8). Hybridizations with a probe covering either the 5′ or 3′ flanking region of *LiMRE11* are shown. (**C**) Growth retardation of promastigotes *MRE11* null mutants. *L. infantum* WT (white circles), *HYG/NEO MRE11^−/−^* (black squares), *HYG/PUR-MRE11* ^WT^ (black triangles), *HYG/PUR-MRE11* ^H210Y^ (inverted white triangles). (**D**) Susceptibility to methylmethane sulfonate (MMS). *L. infantum* WT (white circles), *HYG/NEO MRE11^−/−^* (black squares), *HYG/PUR-MRE11* ^WT^ (black triangles), *HYG/PUR-MRE11* ^H210Y^ (inverted white triangles).

*L. infantum MRE11* null mutant parasites were generated by replacing the entire ORF (*LinJ27.1790*) with genes coding for the neomycin (*NEO*) and hygromycin (*HYG*) phosphotransferases. The two resistant markers were cloned between the 5′- and 3′-*MRE11* flanking regions and targeting constructs were transfected independently in two rounds by electroporation. Southern blot analysis confirmed the homologous chromosomal integration of the two antibiotic markers in the *MRE11* locus (Figures 5A and 5B). Genomic DNAs of the WT and the *HYG/NEO MRE11^−/−^* null mutant were digested with XhoI, transferred onto membranes and hybridized. Hybridization with a probe recognizing the 5′*UTR* region of *MRE11* yielded a 3 kb band in WT cells (Figure 5A and 5B-lane 1) while hybridization with a 3′*UTR* probe generated a 3,4 kb band as expected (Figure 5A and 5B, lane 5). In the *HYG/NEO MRE11^−/−^* strain, replacement of both *MRE11* wild-type alleles by *NEO* and *HYG* led, as expected, to 4,7 kb and 4,9 kb bands respectively, with either *UTR* probes (Figure 5A and 5B, lanes 2 and 6).

It is standard practice to introduce episomal copies of the corresponding wild-type gene in a null mutant background to reverse a potential phenotype. However, we noticed that episomal overexpression of MRE11 as Psp72-α-*PUR*-α-*MRE11^WT^* in WT cells led to a growth defect (Figure S2A). This prompted us to use an alternative to generate revertants. We replaced the *NEO* chromosomal integrated cassette in the MRE11 null mutant by a re-expressing cassette containing either a WT or a mutated allele (H210Y) of *LiMRE11* along with the *PUR* gene in order to generate respectively the *HYG/PUR-MRE11* ^WT^ and *HYG/PUR-MRE11* ^H210Y^ re-expressing add back strains. Hybridization of the DNAs of the add-back strains with a 5′*UTR* probe led, as expected, to a 3 kb band in both strains corresponding to the restoration of a WT allele at the *MRE11* locus, a 4,9 kb band corresponding to the *HYG* chromosomal integration, and a loss of the 4,7 kb-NEO containing band (Figure 5A and 5B-lanes 3 and 4) which was replaced by the *MRE11*-α-*PUR* re-expressing cassette as supported by the hybridization of a 2,7 kb band when using a 3′*UTR* probe (Figure 5A and 5B-lanes 7 and 8). MRE11 expression level was assessed by quantitative real-time RT-PCR in the various cell lines generated. As expected *MRE11* expression level was not detectable in the *MRE11^−/−^* null mutant while it was approximately half the level of the WT in both add back strains, consistent with one new active allele (Figure S3).

A growth defect was observed in *L. infantum HYG/NEO MRE11^−/−^* parasites compared to the WT strain. Promastigotes of the WT strain had a calculated generation time of 12 hours while the *MRE11* null mutant had a generation time of 26 hours. (Figure 5C). Reintroduction of one intact WT or mutated (H210Y) allele of *MRE11* into the chromosomal locus in add back strains partially rescued the growth defect with respective generation time of 15 and 22 hours.(Figure 5C).

Since the MRE11 complex is known to promote repair of DNA double-strand breaks (DSBs) [44], [45], we tested the impact of the *LiMRE11* inactivation using the alkylating damaging agent methyl methanesulphonate (MMS), a compound known to induce DSBs [46]. The *HYG/NEO MRE11^−/−^* cells were significantly more sensitive to MMS compared to both WT and *MRE11* add back re-expressing cells (Figure 5D). As indicated above, intriguingly, overexpressing MRE11 in WT cells led to a growth defect (Figure S2A) but also to a significant increase in MMS sensitivity (Figure S2B). However, episomal overexpression of MRE11^WT^ in *HYG/NEO MRE11* ^−/−^ cells restores the growth defect and MMS susceptibility of the mutant strain to WT levels (Figures S2A and S2B).

### Gene amplification in cells with varying copy of MRE11

**Figure 6:**
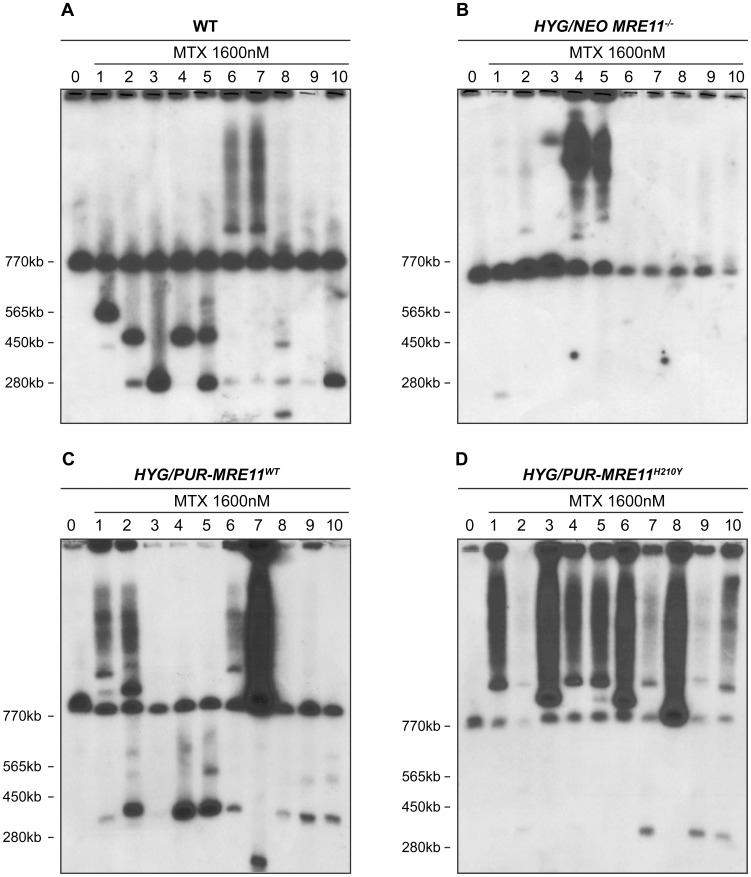
*PTR1* gene amplification of *L. infantum* methotrexate (MTX) resistant cells. *L. infantum* cells were selected for MTX resistance, and their chromosomes were separated by pulsed-field gel electrophoresis using a separation range between 150 kb and 1500 kb, transferred on membranes then hybridized with a *PTR1* probe. MTX-resistant clones resistant to 1600 nM MTX derived from the WT (**A**), the *HYG/NEO MRE11^−/−^* cells (**B**), the *HYG/PUR-MRE11* ^WT^ cells (**C**) and the *HYG/PUR-MRE11* ^H210Y^ cells (**D**). Lanes 0 are parasites without drug selection.

We compared the ability of the *MRE11* null mutants and WT cells to generate extrachromosomal linear amplicons. We selected clones of wild-type cells and of *HYG/NEO MRE11^−/−^* for MTX resistance in a stepwise manner (up to 1600 nM, a 16-fold increase in resistance compared to starting parent cells), a drug known to select for *PTR1* linear DNA amplifications [13], [18], [28]. *Leishmania* chromosomes extracted from ten MTX resistant clones derived from either WT or *HYG/NEO MRE11^−/−^* parasites were separated by pulse field gel electrophoresis (PFGE) and hybridized with a *PTR1* probe. Ethidium bromide stained gels already indicated that some linear amplicons smaller than the smallest chromosome were present in some resistant clones derived from WT but not in the MTX resistant *MRE11^−/−^* mutants (Figure S4). Hybridization data revealed that all ten MTX resistant clones derived from WT cells displayed*PTR1* linear amplicons of varying size of 125 kb, 250 kb, 450 kb and 565 kb (Figure 6A). Clones 6 and 7 also gave rise to *PTR1* circular amplicons, as suggested from the hybridizing smears (Figure 6A). The situation was drastically different in the *HYG/NEO MRE11^−/−^* parasites selected for MTX resistance. We observed only one resistant clone from the *MRE11* null mutant with a faint hybridization signal corresponding to a *PTR1* linear amplification (Figure 6B, clone 1), while a *PTR1* circular amplification was present in clones 4 and 5 derived from the *MRE11^−/−^* mutant (Figure 6B). Clone 3 displayed a hybridization signal at around 1150 kb (Figure 6B) which could correspond to either a very large linear amplicon or to a chromosomal translocation. The difference in formation of linear amplicons between WT and *MRE11^−/−^* null mutant was found to be significant (p<0,01). We also selected the add back strains *HYG/PUR-MRE11* ^WT^ and *HYG/PUR-MRE11* ^H210Y^ for MTX resistance for testing for the specificity of the phenotype and for assessing the role of the MRE11-exonuclease activity in the generation of linear amplicons. While the *MRE11^−/−^* mutants had a decreased capacity to generate linear amplicons after MTX selection (Figure 6B), nine out of ten MTX resistant clones derived from the *HYG/PUR-MRE11* ^WT^ add back strain had *PTR1* linear amplicons (Figure 6C, clones 1, 2, 4–10). Similar to the mutants derived from the wild-type cells (Figure 6A), four different *PTR1* linear amplicons of 125, 250, 450 and 565 kb (Figure 6C) were present and four clones derived from *HYG/PUR-MRE11* ^WT^ had additional *PTR1* circular amplicons (Figure 6C, clones 1, 2, 6 and 7). This phenotype reversion was not observed when the *MRE11^−/−^* cells were complemented with MRE11^WT^ as part of an episomal construct. Clones derived from the latter transfectants and selected for MTX resistance were similar to the *MRE11^−/−^* mutants with no *PTR1* linear amplicons (Figure S5). The results were even more surprising with the MTX resistant clones derived from *HYG/PUR-MRE11* ^H210Y^. Strikingly all mutants had circular amplifications and the *PTR1* hybridization intensity was in general much higher suggesting a higher copy number of the circles. Four clones derived from this add-back revertant also had a *PTR1* linear amplicon (Figure 6D, clones 2, 7, 9, 10).

**Figure 7:**
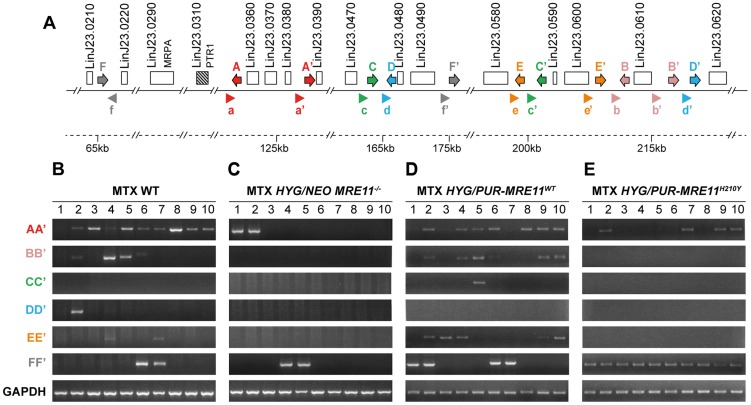
Detection of gene rearrangements leading to *PTR1* containing amplicons using PCR assays. (**A**) Schematic representation of inverted repeated sequences (arrows depicted as A, A′, B, B′, C, C′, D, D′, E, E′) and direct repeats (F, F′) at the *PTR1* chromosomal locus on chromosome 23. Arrowheads (depicted as a, a′, b, b′, c, c′, d, d′, e, e′, f and f′) indicate position and orientation of PCR primers that were used to detect amplicon junctions. (**B–E**) PCR amplification of newly formed amplicon junctions in ten MTX-resistant clones derived either from WT (**B**), *HYG/NEO MRE11^−/−^* (**C**), *HYG/PUR-MRE11* ^WT^ (**D**) and *HYG/PUR-MRE11* ^H210Y^ (**E**).

Previous data has indicated that linear amplicons are constituted of inverted duplications rearranged at the level of IRs with the formation of a new junction that can be amplified by PCR (see Figure 1). The diversity in size of linear amplicons observed in Figure 6A and 6C would suggest that different IRs were used and we tested for the presence of IRs in the chromosome 23 that could lead to PTR1 amplicons with size (125, 250, 450 and 565 kb) consistent with what observed in the blots. We detected a potential of 5 such IRs with size ranging from 440 to 790 bp and with a minimum of 85% identity (Figure 7A), a finding consistent with our demonstration that low copy repeated sequences are widespread throughout the genomes [19]. We performed PCR assays using five different pairs of primers recognizing the five different pairs of IRs under the principle shown in Figure 1. Amplification of the *GAPDH* gene was also done as a control. In the ten MTX resistant clones derived from WT cells and the *HYG/PUR-MRE11^WT^* add back strain, we detected several junctions by PCR (Figure 7B and D). The junction formed following a rearrangement at the level of IRs AA′ was the most frequently observed rearrangement, but junctions BB′, EE′ were also detected frequently while the junctions DD′ and CC′ were detected once in clones derived respectively from the WT or add back strains (Figures 7B and 7D). In clones in which we detected circles by Southern blots (Figure 6), we also obtained a positive signal for junction FF′ where the repeats are in direct orientation (Figure 7). There was a general good agreement between the number of amplicons detected by southern blots and PCR although PCR was more sensitive. For example, we could not detect a linear amplicon in clone 2 in *MRE11^−/−^* (Figure 6B) but it had a positive PCR reaction for the junction AA′ (Figure 7C).

The *MRE11^−/−^* MTX resistant mutants do not have *PTR1* amplification (Figure 6B) and these cells must resist MTX by other means. Several mechanisms of resistance have been described [47], [48] including transport defects and gene amplification. We have carried out transport experiments in the mutants and observed no difference in MTX uptake between the *MRE11^−/−^* and the *MRE11^−/−^* MTX resistant mutants. We also hybridized PFGE blots with a *DHFR-TS* probe and while we failed in detecting circular *DHFR-TS* amplification, we observed an extra high molecular weight band hybridizing to *DHFR-TS* (Figure S6). The exact mechanism leading to this rearrangement is not known but it leads to a 2-fold increase in *DHFR-TS* expression (Figure S7) and it may contribute to MTX resistance.

## Discussion

Gene amplification as part of linear or circular extrachromosomal elements is frequently observed in the parasite *Leishmania* selected for drug resistance or subjected to nutritional stresses [11]. The circles or linear elements are formed at the level of homologous direct or inverted repeats, with more than 2000 repeats of more than 200 bp representing close to 5% of the *Leishmania* genome [19]. The genome of *Leishmania* is continuously being rearranged at the level of these repeats and while each cell has a core genome, they each differ by a complement of circular or linear amplicons. Upon selection the copy number of these elements increases and in the absence of selection the copy number of these elements decreases [19]. It was shown that circular elements are formed by homologous recombination between direct repeated sequences which is catalyzed by the RAD51 recombinase, known to be involved in the homologous recombination process in kinetoplastids [49], [50]. However, the rate of formation of linear amplicons was unchanged in a *RAD51* ^−/−^ mutant and linear amplicons must then be formed through another pathway [19]. We hypothesized that DNA repair proteins with nuclease activities may be involved since genomic DNA must be processed when inverted repeats are annealing for the formation of linear amplicons (Figure 1). We thus focused our efforts on the nuclease MRE11 that is part of the MRN complex [1], [51].

The biochemical characterization of the *Leishmania* MRE11 protein indicated that it has properties similar to other MRE11 orthologues. Indeed, as previously reported for MRE11 homologs in other organisms [42], [51]–[53], it binds preferentially SS and SA DNA structures in competitive assays (Figure 2); it is capable of DNA end resection and exhibited a 3′→5′ exonuclease activity on DS DNA structures, albeit with less effectiveness than the human enzyme (Figure 3). A H210Y mutant abolished its nuclease activity without impairing its DNA binding properties (Figures 2 and 3A). Finally, LiMRE11 recruitment at DNA damage loci was demonstrated in human ATLD cells, an indication that LiMRE11 detects and binds DNA breaks in these cells (Figure 4).

Having established that the *Leishmania* MRE11 protein bears all the hallmarks of the MRE11 family of DNA nucleases, we generated a recombinant parasite where the two alleles were inactivated (Figure 5). These parasites were viable but displayed a growth defect (Figure 5C). The parasites were also more sensitive to the DNA damaging agent MMS (Figure 5D) as also observed for *Trypanosoma brucei* [54]. The growth defect and susceptibility to MMS were reverted when we re-introduced one allele of *MRE11* at its original chromosomal locus (Figures 5C and 5D) or as part of an episomal construct (Figure S2). Surprisingly, the expression of an episomal *MRE11* gene in a WT strain severely impaired its growth rate (Figure S2A) and led to increased sensitivity to MMS (Figure 2). This result strongly suggests that in a wild-type background, the overproduction of MRE11 is somehow affecting the parasite cell growth, a phenomenon usually not observed in other cell types. The expression level of GFP-MRE11 in WT cells was very low, a phenotype also reported in the trypanosomatid parasite *Trypanosoma brucei* [55]. These early divergent eukaryotes may be more sensitive to exonuclease overexpression. Alternatively, overexpression of MRE11 in *Leishmania* may alter more acutely the stoichiometry of interactions with partner proteins such as RAD50 [56] and this could have an impact on cell growth. Interestingly and in support of the above hypothesis, we have shown in an independent study that *RAD50* is essential in *Leishmania* WT cells but its gene can be inactivated in a *MRE11^−/−^* background (Laffitte *et al.*, unpublished data). Possibly that recombination pathways are changed in *MRE11^−/−^* to compensate for the loss of *MRE11*, therefore altering the importance of the MRN complex and its components. Growth delay observed in WT cells overexpressing MRE11 may also relate to the known role of MRE11 in cell cycle regulation [37]. Indeed, MRE11 is involved in control of DNA replication initiation [57] and overexpression of MRE11 in *Leishmania* may have stronger effect on replication of *Leishmania* chromosomes.

Selection for MTX resistance often leads to linear amplifications of *PTR1* in *Leishmania* [13], [14], [28]. We selected wild-type cells, *MRE11^−/−^* null mutants and reverted lines for MTX resistance. Amplified linear *PTR1*-containing amplicons were observed in all the clones derived from the WT strain but in only 1 out of 10 clones derived from the *MRE11^−/−^* null mutant (Figure 6). The capacity to generate circular amplicons was similar in the two different lines (Figure 6A and 6B). This strong phenotype was specific to *MRE11*, as reintroduction of *MRE11* at the original chromosomal locus restored the ability of the parasites to generate *PTR1* linear amplicons upon MTX selection (Figure 6C). *Leishmania* differs from yeast in this process since the MRN complex can prevent palindrome amplification in yeast. This process requires the interaction with the CtIP protein [36], [58] which is absent in *Leishmania* [41], possibly explaining the difference between the two organisms. Two others important parameters to consider are the length of the inverted repeats, which are very long in *Leishmania*, and the length of the sequences between these repeats. Indeed, it was previously suggested in yeast that hairpins with large loops are handled differently than hairpin with smaller loops [59]. This explanation is consistent with our study where IRs are few kb apart (Figure 7A), creating large loops in the hairpin structure, while most of the experiments done in yeast presents IRs closer to each other [58]–[61]. Further experiments could be interesting to determine whether the hairpin strength and structure influences DNA processing by the MRN complex.

The reversion of linear amplicons phenotype is dependent on MRE11 nuclease activity since reintegration of the mutated version MRE11^H210Y^ led to parasites generating more efficiently circular *PTR1* amplicons but not linear ones (Figure 6D). The mutated MRE11 therefore appears to favor homologous recombination in rescued parasites at the level of direct repeated sequences leading to circular amplicons. It is known that MRE11 is involved in initial events of homologous recombination in many organisms [37], [38], [62] and can interact with a number of nucleases and helicases, several of which are encoded in the *L. infantum* genome [41]. We suggest that the *Leishmania* MRE11^H210Y^ is still capable of binding DNA and therefore MRN complex formation is intact, as it was previously suggested in yeast [63]. However, MRE11 lack of nuclease activity possibly makes it a better bait for recruiting HR proteins including RAD51. This facilitated recruitment could be due for example to putative longer association kinetics of the mutated MRE11 to DNA. Alternatively, the inability of LiMRE11^H210Y^ to perform DNA resection may alter the first steps of DNA repair and possibly increase HR proteins recruitment, hence facilitating the formation of circular amplicons. This phenotype is observed in a *MRE11^−/−^* background in which we believe that recombination pathways have changed, possibly for compensating loss of MRE11. Thus, a combination of alterations in recombination pathways along with the mutated MRE11 may be responsible for the phenotype observed. It is salient to reiterate that while the episomal expression of MRE11 in the *MRE11^−/−^* null mutants reverted the growth phenotype and sensitivity to MMS (Figure S2), it did not revert the phenotype of generating linear amplicons upon MTX selection (Figure S5). This is a further demonstration of the importance of a suitable level of expression to recover proper MRE11 functions.

We have shown that gene rearrangements are continuously taking place at the level or repeated sequences, and that these rearrangements can be highlighted by PCR assays. Using PCR, we have shown that the *PTR1* linear amplicons are generated at the level of 5 different inverted repeats, indicating that different rearrangements led to the linear amplicons (Figure 7). The IRs most frequently used are AA′, BB′ and EE′ and these are relatively close to one another (10 kb between A and A′ as well as between E and E′, 3 kb between B and B′) while IRs CC′ and DD′ are further apart (respectively 38 and 53 kb) and used only once. This suggests that the length of the intervening sequences between the IRs may impact the rate of annealing of the IRs and the rearrangements leading to linear amplicons. Few amplicons detected by southern blot were not observed by PCR, suggesting that either smaller inverted repeats were used or secondary rearrangements occurred. Our bioinformatics screen has revealed only one direct repeat that could entertain *PTR1* circular amplification and indeed the PCR assay has revealed that in every cell in which a circular amplicon was observed in Figure 6, we observed a positive PCR signal indicative of recombination between direct repeat sequences FF′ (Figure 7).

Because of its lack of control at the level of transcription initiation, *Leishmania* is likely to use several mechanisms to regulate its expression. We have suggested that gene rearrangements leading to copy number variation is one such mechanism. Indeed the whole *Leishmania* genome is continuously and stochastically rearranged at the level of repeated sequences. We have shown that there are at least two pathways of rearrangement. One leads to circles after recombination between two direct repeated sequences and this requires RAD51. Here we have shown that linear amplicons, formed at the annealing of two IRs, is facilitated by the presence of an active MRE11. We proposed that double-strand breaks (see Figure 1) would be necessary, although this will require experimental validation, which may be challenging in *Leishmania* as they are no suitable inducible systems. Gene rearrangement is one main mechanism of resistance in *Leishmania* and a further understanding of the proteins involved in gene rearrangements may provide a strategy to circumvent the emergence of drug resistance.

## Materials and Methods

### Strains, culture conditions

Promastigotes of *Leishmania infantum* (MHOM/MA/67/ITMAP-263) and all recombinants were grown in SDM-79 medium at 25°C supplemented with 10% fetal bovine serum, 5 µg/ml of hemin at pH 7.0. Independent clones of all cells generated in this study were selected for methotrexate (MTX) resistance, using a stepwise selection starting from an EC_50_ of 100 nM up to 1600 nM of MTX. All chemical reagents were purchased from Sigma-Aldrich unless specified and were of the highest grades.

### DNA constructs and protein purification

The *L. infantum MRE11* gene (*LinJ.27.1790*) was amplified by PCR using primers 1 and 2 (Table S1) on genomic DNA template and cloned in a modified pFASTBAC1 plasmid (Invitrogen) [64] encoding the glutathione-S-transferase tag (GST) at the N-terminus of MRE11 and a 10-histidine tag at its C-terminus for protein purification. Site-directed mutagenesis (Stratagene, Quickchange) was used to generate the LiMRE11 mutant H210Y using primers 15 and 16 (Table S1). The LiMRE11^WT^ protein and the mutated version LiMRE11^H210Y^ were purified from baculovirus-infected SF9 cells and the GST tag was removed by PreScission cleavage as described in [64]. Full-length human MRE11 cDNAs cloned in pFASTBAC were generously provided by Tanya Paull (University of Texas, Austin). Primers 22 and 23 (Table S1) were used for site-directed mutagenesis (Stratagene, Quickchange) to generate the human MRE11 mutant H217Y. Proteins hMRE11^WT^ and hMRE11^H217Y^ were purified as described [65]. Full-length human MRE11 cDNAs cloned in pEYFP-C1 (Clontech) was generously provided by John Petrini (University of California, San Francisco). The fluorescence observed with pEYFP-C1 is equivalent to that from pEGFP-C1. The *L. infantum* gene *LiMRE11^WT^* was cloned in pEGFP-C1 plasmid (Clontech, encoding a GFP tag located at the N-terminus) for FRAP analysis.

### DNA binding assays

DNA substrates were made by the annealing of the ^32^P-labelled primer 21 with either primer 17 for double-stranded DNA substrate (DS) or primer 20 for splayed arm (SA) (Table S1). Reactions (10 µL) contained 25 nM of ^32^P-labeled DNA oligonucleotides with the indicated concentration of proteins (see Figure 2) in MOPS buffer (25 mM MOPS (morpholine-propanesulfonic acid) pH 7.0, 0,2% tween-20, 2 mM CaCl_2_ and 2 mM DTT). After 15 minutes of incubation at 37°C, reactions were fixed at 37°C during 15 minutes with 0.2% glutaraldehyde. Samples were loaded onto a 8% TBE 1× acrylamide gel, run at 150 V for 1h30, followed by autoradiography.

### Exonuclease assays

Exonuclease assays were performed in MOPS/EXO buffer (25 mM MOPS (morpholine-propanesulfonic acid) pH 7.0, 60 mM KCl, 0.2% tween-20, 2 mM DTT, 2 mM ATP, 5 mM MnCl_2_). Double-stranded DNA substrate (DS) was generated as stated above. The indicated concentration of proteins (see Figure 3) were incubated in MOPS/EXO buffer with 200 nM of ^32^P-labeled DNA for 30 minutes at 37°C, followed by deproteinization in one-fifth volume of stop buffer (20 mM Tris-Cl pH 7.5 and 2 mg/mL proteinase K) for 30 minutes at 37°C. The reactions were boiled during 5 minutes at 95°C after the addition of formamide blue (50% final) then loaded on 8% acrylamide/urea gels. Gels were run at 75W for 60 minutes, dried onto DE81 filter paper, followed by autoradiography. For exonuclease assay on different DNA substrates, ^32^P-labeled oligonucleotide 21 (Table S1) was labeled at the 5′-end (using the terminal transferase and the New England Biolabs protocol) and hybridized to primers 17, 18 and 19 (Table S1).

### FRAP analysis

ATLD human cells (kindly obtained from Yossi Shiloh, University of Tel Aviv, Israël) were maintained in DMEM medium supplemented with 20% fetal bovine serum and 1% penicillin/streptomycin (Life Technologies). ATLD cells were transfected by electroporation with 50 µg of *LiMRE11-GFP* or *hMRE11-GFP* DNA constructs. After 16 hours, we performed Fluorescence recovery after photobleaching (FRAP) analysis. Briefly, fluorescence was monitored on a Leica TCS SP5 II confocal microscope and laser-induced DNA damage was created using a 405-nm UV laser. Visualization of GFP fluorescence within the micro-irradiated nuclear region was achieved using a 488 nm excitation filter and a 63× objective. Background and photo-bleaching corrections were applied to each dataset using the Volocity-software.

### Generation of *LiMRE11* (*LinJ27.1790*) null mutant cells

The *L. infantum MRE11* null mutant (*MRE11^−/−^*) cells were obtained by targeted gene replacement. *MRE11* flanking regions were amplified from *L. infantum* wild-type genomic DNA and fused to both neomycin phosphotransferase (*NEO*) and hygromycin phosphotransferase (*HYG*) genes using a PCR fusion based-method as described previously [66]. Briefly, 5′UTR of *MRE11* was amplified using primers 3 and 4 for the *NEO* cassette and primers 3 and 5 for the *HYG* cassette. The *NEO* gene was amplified with primers 7 and 10 and the *HYG* gene with primers 8 and 11. 3′UTR of *MRE11* was amplified using primers 13 and 14 for both inactivation cassettes (see primer sequences in Table S1). At least 3 µg of the *5′UTR-NEO-3′UTR* and *5′UTR-HYG-3′UTR* linear fragments were successively transfected by electroporation (as described in [67]) into *L. infantum* WT to replace both *MRE11* alleles. Recombinants were selected in the presence of 300 µg/ml of hygromycin B (New England Biolabs, Beverly, MA, USA) and 40 µg/ml of G418 (Geneticin; Sigma-Aldrich). After 4–5 passages, cells resistant to the drug selection were cloned in SDM-Agar plates (1%) in the presence of antibiotics at the same concentrations. Ten clones of each recombinant were picked up after 10 days and used for further analysis.

### Re-expression of MRE11 in null mutant cells

A re-expression cassette, *5′UTR-MRE11*-*α-PUR*-*3′UTR* was designed to reintroduce *MRE11* into its original chromosomal locus in the *HYG/NEO MRE11^−/−^* null mutant. Briefly, this cassette was obtained by PCR of the *PUR* gene using primers 9 and 12 on the plasmid template Psp72-α-PUR-α [68] encoding the puromycin acetyltransferase marker. This fragment was fused to the 5′UTR and coding sequences of *MRE11* (amplified using primers 3 and 6) and 3′UTR fragments (amplified using primers 13 and 14 in Table S1). The cassette was then transfected by electroporation in the *L. infantum HYG/NEO MRE11^−/−^* parasites to replace the *NEO* allele and recombinants were selected with 100 µg/ml of puromycin (Sigma–Aldrich) and 300 µg/ml of hygromycin B (New England Biolabs). The same strategy was used to introduce *MRE11* containing the mutation H210Y in the *HYG/NEO MRE11^−/−^* strain. The *MRE11* ORF was also cloned in the episomal plasmid Psp72-α-puro-α, transfected in *L. infantum* WT and *HYG/NEO MRE11^−/−^* parasites, and stable transfectants were selected with 100 µg/ml of puromycin.

### Southern blot analyses

*MRE11* allele replacement was confirmed by Southern blot analyses. Genomic DNAs from clones were isolated using DNAzol as recommended by the manufacturer (Invitrogen). Digested genomic DNAs or separated chromosomes were subjected to Southern blot hybridization with [α-^32^P]dCTP-labeled DNA probes according to standard protocols [69]. All probes were obtained by PCR (see primers in Table S1) from *L. infantum* genomic DNAs.

### Quantitative real-time RT-PCR

RNAs were extracted using RNeasy plus mini kit (Sigma) according to the manufacturer recommendations. The cDNA was synthesized using Oligo dT_12–18_ and SuperScript II RNase H-Reverse Transcriptase (Invitrogen) and amplified in SYBR Green Supermix (Bio-Rad) using a rotator thermocycler Rotor Gene (RG 3000, Corbett Research). The expression level was derived from three technical and three biological replicates and was normalized to constitutively expressed mRNA encoding glyceraldehyde-3-phosphate dehygrogenase (*GAPDH, LinJ.36.2480*). The sequences of the primers used in this assay are listed in Table S1.

### Methylmethane sulfonate (MMS) assays

*L. infantum* WT, *HYG/NEO MRE11^−/−^*, MRE11 re-expressing cells (*HYG/PUR-MRE11* ^WT^ and *HYG/PUR-MRE11* ^H210Y^), WT Psp72-α-puro-α-MRE11 and *HYG/NEO MRE11^−/−^* Psp72-α-puro-α-*MRE11* were resuspended at a concentration of 5×10^6^ cells/ml and exposed to increasing doses of MMS (Sigma–Aldrich). Cells were counted after 72 h and reported in survival rate.

### Pulsed-field gel electrophoresis

Intact chromosomes were prepared from *L. infantum* promastigotes harvested from log phase cultures, washed once in 1× Hepes-NaCl buffer then lysed in situ in 1% low melting agarose plugs. Briefly, cells were resuspended in HEPES-NaCl buffer at a density of 5×10^7^ cells/ml and mixed with an equal volume of low melting-point agarose (Invitrogen). Cells were then lysed overnight at 50°C in lysis buffer (0.5M ethylenediaminetetraacetic acid (EDTA) pH 9.5, 1% sodium dodecyl sulfate (SDS), 350 ug/ml proteinase K). *Leishmania* intact chromosomes were separated in 1× TBE buffer (from 10× TBE: 1M Tris, 1M Acid boric, 0,02M EDTA) by Pulsed-Field Gel Electrophoresis (PFGE) using a Bio-Rad CHEF-DR III apparatus at 5 V/cm and a 120°C separation angle as described previously [70]. The range of chromosome separation was between 150 and 1500 kb.

### Bioinformatics analyses and primer design

Repeated intergenic sequences were already characterized [19]. Primers (see Table S1) used to detect new junctions created by amplicon formation (Figure 1) were designed for all putative recombination/annealing events between repeated sequences. Primers were chosen within 150 nucleotides from the repeated sequences with their orientation shown in Figure 1. Optimal primer length was 20 nucleotides and optimal melting temperature (Tm) was 64°C.

### DNA preparation for semi quantitative qPCR assays

Late log phase promastigotes (30 ml) were pelleted at 3000 rpm for 5 minutes and pellets were washed once with 1× HEPES-NaCl, resuspended in suspension buffer (100 mM EDTA, 100 mM NaCl, 10 mM Tris pH 8.0), then lysed in 1% SDS and 50 µg/ml proteinase K at 37°C for 2 hours. Genomic DNAs were extracted with 1 volume phenol, precipitated with 2 volume 99% ethanol, washed with 70% ethanol twice and dissolved in 1 ml 1× TE buffer. RNAse A (Qiagen) was added at 20 µg/ml and DNAs were incubated at 37°C for 30 minutes, followed by the addition of 50 µg/ml of proteinase K and 0.1% SDS at 37°C for 30 minutes. DNAs were extracted with 1 volume of phenol, precipitated and washed in ethanol, and dissolved in DNase free-water (Millipore) at 37°C overnight.

PCR reaction mixtures consisted of 100 ng of genomic DNA isolated as described above, 1 µl of forward and reverse primers at 100 µM (Table S1), 0.5 µl dNTP mix at 10 mM, 1.25 U of FastStart Taq DNA polymerase (Roche), 1× PCR buffer+MgCl_2_ and 1.25 µl BSA at 66 mg/ml. The total reaction mixture was made up to 25 µl by addition of the genomic DNA. For each PCR reaction, the number of cycles was optimized to prevent saturation of the amplification. Saturation of band intensities of the amplified PCR products was determined using the AlphaImager 2000 software. The housekeeping chromosomal gene *GAPDH* (*LinJ36.2480*) was used as an internal control (primers pair gg' in Table S1) to normalize the amount of DNA loaded in each reaction.

## Supporting Information

Figure S1 Alignment with ClustalX of MRE11 protein sequences from human, *Saccharomyces cerevisiae* and *Leishmania infantum*. Blue represents residues: ACFIMVW; dark blue: HY; pink: ED; green: NQST; yellow: P; orange: G; coral: KR; “*” indicates position which have a single fully conserved residue; “:” indicates a strong group of conserved amino acids; “.” indicates a weaker group of conserved amino acids.
(TIF)

Figure S2 Episomal expression of MRE11 in WT cells and in *MRE11^−/−^ L. infantum*. Overexpression of LiMRE11^WT^ derived from an episomal construct in *L. infantum* WT is impairing cell growth (**A**) and increases sensitivity to MMS (**B**). The episomal expression of MRE11, however rescued the growth (**A**) and sensitivity phenotypes (**B**) of the *MRE11^−/−^* parasites. *Leishmania infantum* WT strain (white circles); *L. infantum HYG/NEO MRE11^−/−^* (black squares); *L. infantum* WT in which an episomal expressing LiMRE11^WT^ construct has been transfected (white diamonds); strain *HYG/NEO MRE11^−/−^* in which an episomal expressing LiMRE11^WT^ construct has been transfected (black X).
(TIF)

Figure S3 *MRE11* RNA expression in *Leishmania* cells. *MRE11* mRNA levels were analyzed by quantitative real-time RT-PCR. The *MRE11* RNA expression ratios were normalized to *GAPDH* expression.
(TIF)

Figure S4 MTX-resistant clones derived from the WT and the *HYG/PUR-MRE11* ^WT^ strains display DNA bands smaller than the smallest genomic chromosome (indicated by arrows) that correspond to linear amplicons of 300 kb. *L. infantum* chromosomes were separated by pulsed-field gel electrophoresis using a separation range between 150 kb and 1500 kb and incubated with ethidium bromide. MTX-resistant clones resistant to 1600 nM MTX derived from the WT (**A**), the *HYG/NEO MRE11^−/−^* cells (**B**), the *HYG/PUR-MRE11* ^WT^ cells (**C**) and the *HYG/PUR-MRE11* ^H210Y^ cells (**D**). Lanes 0 are parasites without drug selection.
(TIF)

Figure S5 Lack of *PTR1* gene amplification in *L. infantum MRE11^−/−^* cells complemented with an episomal *MRE11* selected for methotrexate (MTX) resistance. *HYG/NEO MRE11^−/−^* Psp72-α-puro-α-*MRE11* cells were selected for MTX resistance, and their chromosomes were separated by pulsed-field gel electrophoresis using a separation range between 150 kb and 1500 kb. The blot was transferred on membranes and hybridized with a *PTR1* probe. Lanes 0 are parasites without drug selection.
(TIF)

Figure S6 *DHFR-TS* gene rearrangement of *L. infantum MRE11^−/−^* cells selected for methotrexate (MTX) resistance. *L. infantum* cells were selected for MTX resistance, and their chromosomes were separated by pulsed-field gel electrophoresis using a separation range between 150 kb and 1500 kb, transferred on membranes then hybridized with a *DHFR-TS* probe. MTX-resistant clones resistant to 1600 nM MTX derived from the WT (**A**), the *HYG/NEO MRE11^−/−^* cells (**B**), the *HYG/PUR-MRE11* ^WT^ cells (**C**) and the *HYG/PUR-MRE11* ^H210Y^ cells (**D**). Lanes 0 are parasites without drug selection.
(TIF)

Figure S7 *DHFR-TS* RNA expression in *MRE11^−/−^* cells selected for MTX resistance. The RNAs derived from the *MRE11^−/−^* and from five *MRE11^−/−^* methotrexate resistant clones were analyzed by quantitative real-time RT-PCR. The *DHFR-TS* RNA expression ratios were normalized to *GAPDH* expression.
(TIF)

Table S1 Primers used in this study were designed using PrimerQuest software.
(DOCX)

## References

[1] Handman E (2001) Leishmaniasis: current status of vaccine development. Clin Microbiol Rev 14: 229–243. 11292637 10.1128/CMR.14.2.229-243.2001 PMC88972

[2] Murray HW, Berman JD, Davies CR, Saravia NG (2005) Advances in leishmaniasis. Lancet 366: 1561–1577. 16257344 10.1016/S0140-6736(05)67629-5

[3] Lira R, Sundar S, Makharia A, Kenney R, Gam A, et al (1999) Evidence that the high incidence of treatment failures in Indian kala-azar is due to the emergence of antimony-resistant strains of Leishmania donovani. J Infect Dis 180: 564–567. 10395884 10.1086/314896

[4] Sundar S, More DK, Singh MK, Singh VP, Sharma S, et al (2000) Failure of pentavalent antimony in visceral leishmaniasis in India: report from the center of the Indian epidemic. Clin Infect Dis 31: 1104–1107. 11049798 10.1086/318121

[5] Hadighi R, Mohebali M, Boucher P, Hajjaran H, Khamesipour A, et al (2006) Unresponsiveness to Glucantime treatment in Iranian cutaneous leishmaniasis due to drug-resistant Leishmania tropica parasites. PLoS Med 3: e162. 16605301 10.1371/journal.pmed.0030162 PMC1435779

[6] Rojas R, Valderrama L, Valderrama M, Varona MX, Ouellette M, et al (2006) Resistance to antimony and treatment failure in human Leishmania (Viannia) infection. J Infect Dis 193: 1375–1383. 16619185 10.1086/503371

[7] Torres DC, Adaui V, Ribeiro-Alves M, Romero GA, Arevalo J, et al (2010) Targeted gene expression profiling in Leishmania braziliensis and Leishmania guyanensis parasites isolated from Brazilian patients with different antimonial treatment outcomes. Infect Genet Evol 10: 727–733. 20478409 10.1016/j.meegid.2010.05.006

[8] Campbell DA, Thomas S, Sturm NR (2003) Transcription in kinetoplastid protozoa: why be normal? Microbes Infect 5: 1231–1240. 14623019 10.1016/j.micinf.2003.09.005

[9] Haile S, Papadopoulou B (2007) Developmental regulation of gene expression in trypanosomatid parasitic protozoa. Curr Opin Microbiol 10: 569–577. 18177626 10.1016/j.mib.2007.10.001

[10] Martinez-Calvillo S, Vizuet-de-Rueda JC, Florencio-Martinez LE, Manning-Cela RG, Figueroa-Angulo EE (2010) Gene expression in trypanosomatid parasites. J Biomed Biotechnol 2010: 525241. 20169133 10.1155/2010/525241 PMC2821653

[11] Beverley SM (1991) Gene amplification in Leishmania. Annu Rev Microbiol 45: 417–444. 1741620 10.1146/annurev.mi.45.100191.002221

[12] Borst P, Ouellette M (1995) New mechanisms of drug resistance in parasitic protozoa. Annu Rev Microbiol 49: 427–460. 8561467 10.1146/annurev.mi.49.100195.002235

[13] Grondin K, Kundig C, Roy G, Ouellette M (1998) Linear amplicons as precursors of amplified circles in methotrexate-resistant Leishmania tarentolae. Nucleic Acids Res 26: 3372–3378. 9649621 10.1093/nar/26.14.3372 PMC147699

[14] Ubeda JM, Legare D, Raymond F, Ouameur AA, Boisvert S, et al (2008) Modulation of gene expression in drug resistant Leishmania is associated with gene amplification, gene deletion and chromosome aneuploidy. Genome Biol 9: R115. 18638379 10.1186/gb-2008-9-7-r115 PMC2530873

[15] Leprohon P, Legare D, Raymond F, Madore E, Hardiman G, et al (2009) Gene expression modulation is associated with gene amplification, supernumerary chromosomes and chromosome loss in antimony-resistant Leishmania infantum. Nucleic Acids Res 37: 1387–1399. 19129236 10.1093/nar/gkn1069 PMC2655676

[16] Downing T, Imamura H, Decuypere S, Clark TG, Coombs GH, et al (2011) Whole genome sequencing of multiple Leishmania donovani clinical isolates provides insights into population structure and mechanisms of drug resistance. Genome Res 21: 2143–2156. 22038251 10.1101/gr.123430.111 PMC3227103

[17] Ouellette M, Hettema E, Wust D, Fase-Fowler F, Borst P (1991) Direct and inverted DNA repeats associated with P-glycoprotein gene amplification in drug resistant Leishmania. EMBO J 10: 1009–1016. 1672636 10.1002/j.1460-2075.1991.tb08035.x PMC452745

[18] Grondin K, Roy G, Ouellette M (1996) Formation of extrachromosomal circular amplicons with direct or inverted duplications in drug-resistant Leishmania tarentolae. Mol Cell Biol 16: 3587–3595. 8668175 10.1128/mcb.16.7.3587 PMC231354

[19] Ubeda JM, Raymond F, Mukherjee A, Plourde M, Gingras H, et al (2014) Genome-wide stochastic adaptive DNA amplification at direct and inverted DNA repeats in the parasite leishmania. PLoS Biol 12: e1001868. 24844805 10.1371/journal.pbio.1001868 PMC4028189

[20] Haimeur A, Brochu C, Genest P, Papadopoulou B, Ouellette M (2000) Amplification of the ABC transporter gene PGPA and increased trypanothione levels in potassium antimonyl tartrate (SbIII) resistant Leishmania tarentolae. Mol Biochem Parasitol 108: 131–135. 10802326 10.1016/s0166-6851(00)00187-0

[21] Coderre JA, Beverley SM, Schimke RT, Santi DV (1983) Overproduction of a bifunctional thymidylate synthetase-dihydrofolate reductase and DNA amplification in methotrexate-resistant Leishmania tropica. Proc Natl Acad Sci U S A 80: 2132–2136. 6572966 10.1073/pnas.80.8.2132 PMC393771

[22] Beverley SM, Coderre JA, Santi DV, Schimke RT (1984) Unstable DNA amplifications in methotrexate-resistant Leishmania consist of extrachromosomal circles which relocalize during stabilization. Cell 38: 431–439. 6467372 10.1016/0092-8674(84)90498-7

[23] Kundig C, Leblanc E, Papadopoulou B, Ouellette M (1999) Role of the locus and of the resistance gene on gene amplification frequency in methotrexate resistant Leishmania tarentolae. Nucleic Acids Res 27: 3653–3659. 10471733 10.1093/nar/27.18.3653 PMC148619

[24] Bello AR, Nare B, Freedman D, Hardy L, Beverley SM (1994) PTR1: a reductase mediating salvage of oxidized pteridines and methotrexate resistance in the protozoan parasite Leishmania major. Proc Natl Acad Sci U S A 91: 11442–11446. 7972081 10.1073/pnas.91.24.11442 PMC45247

[25] Wang J, Leblanc E, Chang CF, Papadopoulou B, Bray T, et al (1997) Pterin and folate reduction by the Leishmania tarentolae H locus short-chain dehydrogenase/reductase PTR1. Arch Biochem Biophys 342: 197–202. 9186479 10.1006/abbi.1997.0126

[26] Hightower RC, Ruiz-Perez LM, Wong ML, Santi DV (1988) Extrachromosomal elements in the lower eukaryote Leishmania. J Biol Chem 263: 16970–16976. 3182826

[27] White TC, Fase-Fowler F, van Luenen H, Calafat J, Borst P (1988) The H circles of Leishmania tarentolae are a unique amplifiable system of oligomeric DNAs associated with drug resistance. J Biol Chem 263: 16977–16983. 3182827

[28] Papadopoulou B, Roy G, Ouellette M (1993) Frequent amplification of a short chain dehydrogenase gene as part of circular and linear amplicons in methotrexate resistant Leishmania. Nucleic Acids Res 21: 4305–4312. 8414986 10.1093/nar/21.18.4305 PMC310065

[29] Chiquero MJ, Olmo A, Navarro P, Ruiz-Perez LM, Castanys S, et al (1994) Amplification of the H locus in Leishmania infantum. Biochim Biophys Acta 1227: 188–194. 7986827 10.1016/0925-4439(94)90094-9

[30] Butler DK, Yasuda LE, Yao MC (1995) An intramolecular recombination mechanism for the formation of the rRNA gene palindrome of Tetrahymena thermophila. Mol Cell Biol 15: 7117–7126. 8524279 10.1128/mcb.15.12.7117 PMC230967

[31] Okuno Y, Hahn PJ, Gilbert DM (2004) Structure of a palindromic amplicon junction implicates microhomology-mediated end joining as a mechanism of sister chromatid fusion during gene amplification. Nucleic Acids Res 32: 749–756. 14757839 10.1093/nar/gkh244 PMC373360

[32] VanHulle K, Lemoine FJ, Narayanan V, Downing B, Hull K, et al (2007) Inverted DNA repeats channel repair of distant double-strand breaks into chromatid fusions and chromosomal rearrangements. Mol Cell Biol 27: 2601–2614. 17242181 10.1128/MCB.01740-06 PMC1899885

[33] Rosenberg SM, Shee C, Frisch RL, Hastings PJ (2012) Stress-induced mutation via DNA breaks in Escherichia coli: a molecular mechanism with implications for evolution and medicine. Bioessays 34: 885–892. 22911060 10.1002/bies.201200050 PMC3533179

[34] Lin CT, Lin WH, Lyu YL, Whang-Peng J (2001) Inverted repeats as genetic elements for promoting DNA inverted duplication: implications in gene amplification. Nucleic Acids Res 29: 3529–3538. 11522822 10.1093/nar/29.17.3529 PMC55881

[35] Rattray AJ, McGill CB, Shafer BK, Strathern JN (2001) Fidelity of mitotic double-strand-break repair in Saccharomyces cerevisiae: a role for SAE2/COM1. Genetics 158: 109–122. 11333222 10.1093/genetics/158.1.109 PMC1461648

[36] Tanaka H, Yao MC (2009) Palindromic gene amplification–an evolutionarily conserved role for DNA inverted repeats in the genome. Nat Rev Cancer 9: 216–224. 19212324 10.1038/nrc2591

[37] Assenmacher N, Hopfner KP (2004) MRE11/RAD50/NBS1: complex activities. Chromosoma 113: 157–166. 15309560 10.1007/s00412-004-0306-4

[38] Stracker TH, Petrini JH (2011) The MRE11 complex: starting from the ends. Nat Rev Mol Cell Biol 12: 90–103. 21252998 10.1038/nrm3047 PMC3905242

[39] Shibata A, Moiani D, Arvai AS, Perry J, Harding SM, et al (2014) DNA double-strand break repair pathway choice is directed by distinct MRE11 nuclease activities. Mol Cell 53: 7–18. 24316220 10.1016/j.molcel.2013.11.003 PMC3909494

[40] Mimitou EP, Symington LS (2011) DNA end resection—unraveling the tail. DNA Repair (Amst) 10: 344–348. 21227759 10.1016/j.dnarep.2010.12.004 PMC3046306

[41] Genois MM, Paquet ER, Laffitte MC, Maity R, Rodrigue A, et al (2014) DNA repair pathways in trypanosomatids: from DNA repair to drug resistance. Microbiol Mol Biol Rev 78: 40–73. 24600040 10.1128/MMBR.00045-13 PMC3957735

[42] Paull TT, Gellert M (2000) A mechanistic basis for Mre11-directed DNA joining at microhomologies. Proc Natl Acad Sci U S A 97: 6409–6414. 10823903 10.1073/pnas.110144297 PMC18616

[43] Stewart GS, Maser RS, Stankovic T, Bressan DA, Kaplan MI, et al (1999) The DNA double-strand break repair gene hMRE11 is mutated in individuals with an ataxia-telangiectasia-like disorder. Cell 99: 577–587. 10612394 10.1016/s0092-8674(00)81547-0

[44] Dolganov GM, Maser RS, Novikov A, Tosto L, Chong S, et al (1996) Human Rad50 is physically associated with human Mre11: identification of a conserved multiprotein complex implicated in recombinational DNA repair. Mol Cell Biol 16: 4832–4841. 8756642 10.1128/mcb.16.9.4832 PMC231485

[45] Bressan DA, Olivares HA, Nelms BE, Petrini JH (1998) Alteration of N-terminal phosphoesterase signature motifs inactivates Saccharomyces cerevisiae Mre11. Genetics 150: 591–600. 9755192 10.1093/genetics/150.2.591 PMC1460356

[46] Strauss B, Wahl R (1964) The presence of breaks in the deoxyribonucleic acid of *Bacillus subtilis* treated *in vivo* with the alkylating agent, methylmethanesulfonate. Biochimica et Biophysica Acta (BBA)-Specialized Section on Nucleic Acids and Related Subjects 80: 116–126.

[47] Nare B, Luba J, Hardy LW, Beverley S (1997) New approaches to Leishmania chemotherapy: pteridine reductase 1 (PTR1) as a target and modulator of antifolate sensitivity. Parasitology 114 Suppl: S101–110. 9309772

[48] Ouellette M, Drummelsmith J, El-Fadili A, Kundig C, Richard D, et al (2002) Pterin transport and metabolism in Leishmania and related trypanosomatid parasites. Int J Parasitol 32: 385–398. 11849635 10.1016/s0020-7519(01)00346-0

[49] McCulloch R, Barry JD (1999) A role for RAD51 and homologous recombination in Trypanosoma brucei antigenic variation. Genes Dev 13: 2875–2888. 10557214 10.1101/gad.13.21.2875 PMC317127

[50] Genois MM, Mukherjee A, Ubeda JM, Buisson R, Paquet E, et al (2012) Interactions between BRCA2 and RAD51 for promoting homologous recombination in Leishmania infantum. Nucleic Acids Res 40: 6570–6584. 22505581 10.1093/nar/gks306 PMC3413117

[51] Wen Q, Scorah J, Phear G, Rodgers G, Rodgers S, et al (2008) A mutant allele of MRE11 found in mismatch repair-deficient tumor cells suppresses the cellular response to DNA replication fork stress in a dominant negative manner. Mol Biol Cell 19: 1693–1705. 18256278 10.1091/mbc.E07-09-0975 PMC2291432

[52] Lee JH, Ghirlando R, Bhaskara V, Hoffmeyer MR, Gu J, et al (2003) Regulation of Mre11/Rad50 by Nbs1: effects on nucleotide-dependent DNA binding and association with ataxia-telangiectasia-like disorder mutant complexes. J Biol Chem 278: 45171–45181. 12966088 10.1074/jbc.M308705200

[53] Yu Z, Vogel G, Coulombe Y, Dubeau D, Spehalski E, et al (2012) The MRE11 GAR motif regulates DNA double-strand break processing and ATR activation. Cell Res 22: 305–320. 21826105 10.1038/cr.2011.128 PMC3271587

[54] Robinson NP, McCulloch R, Conway C, Browitt A, Barry JD (2002) Inactivation of Mre11 does not affect VSG gene duplication mediated by homologous recombination in Trypanosoma brucei. J Biol Chem 277: 26185–26193. 12011090 10.1074/jbc.M203205200

[55] Tan KS, Leal ST, Cross GA (2002) Trypanosoma brucei MRE11 is non-essential but influences growth, homologous recombination and DNA double-strand break repair. Mol Biochem Parasitol 125: 11–21. 12467970 10.1016/s0166-6851(02)00165-2

[56] Hopfner KP, Karcher A, Craig L, Woo TT, Carney JP, et al (2001) Structural biochemistry and interaction architecture of the DNA double-strand break repair Mre11 nuclease and Rad50-ATPase. Cell 105: 473–485. 11371344 10.1016/s0092-8674(01)00335-x

[57] Olson E, Nievera CJ, Liu E, Lee AY, Chen L, et al (2007) The Mre11 complex mediates the S-phase checkpoint through an interaction with replication protein A. Mol Cell Biol 27: 6053–6067. 17591703 10.1128/MCB.00532-07 PMC1952149

[58] Lobachev KS, Gordenin DA, Resnick MA (2002) The Mre11 complex is required for repair of hairpin-capped double-strand breaks and prevention of chromosome rearrangements. Cell 108: 183–193. 11832209 10.1016/s0092-8674(02)00614-1

[59] Zhang Y, Saini N, Sheng Z, Lobachev KS (2013) Genome-wide screen reveals replication pathway for quasi-palindrome fragility dependent on homologous recombination. PLoS Genet 9: e1003979. 24339793 10.1371/journal.pgen.1003979 PMC3855049

[60] Butler DK, Yasuda LE, Yao MC (1996) Induction of large DNA palindrome formation in yeast: implications for gene amplification and genome stability in eukaryotes. Cell 87: 1115–1122. 8978615 10.1016/s0092-8674(00)81805-x

[61] Brewer BJ, Payen C, Raghuraman MK, Dunham MJ (2011) Origin-dependent inverted-repeat amplification: a replication-based model for generating palindromic amplicons. PLoS Genet 7: e1002016. 21437266 10.1371/journal.pgen.1002016 PMC3060070

[62] Lamarche BJ, Orazio NI, Weitzman MD (2010) The MRN complex in double-strand break repair and telomere maintenance. FEBS Lett 584: 3682–3695. 20655309 10.1016/j.febslet.2010.07.029 PMC2946096

[63] Krogh BO, Llorente B, Lam A, Symington LS (2005) Mutations in Mre11 phosphoesterase motif I that impair Saccharomyces cerevisiae Mre11-Rad50-Xrs2 complex stability in addition to nuclease activity. Genetics 171: 1561–1570. 16143598 10.1534/genetics.105.049478 PMC1456084

[64] Maity R, Pauty J, Krietsch J, Buisson R, Genois MM, et al (2013) GST-His purification: a two-step affinity purification protocol yielding full-length purified proteins. J Vis Exp e50320. 24193370 10.3791/50320 PMC3964817

[65] Dery U, Coulombe Y, Rodrigue A, Stasiak A, Richard S, et al (2008) A glycine-arginine domain in control of the human MRE11 DNA repair protein. Mol Cell Biol 28: 3058–3069. 18285453 10.1128/MCB.02025-07 PMC2293076

[66] Moreira W, Leblanc E, Ouellette M (2009) The role of reduced pterins in resistance to reactive oxygen and nitrogen intermediates in the protozoan parasite Leishmania. Free Radic Biol Med 46: 367–375. 19022374 10.1016/j.freeradbiomed.2008.10.034

[67] Papadopoulou B, Roy G, Ouellette M (1992) A novel antifolate resistance gene on the amplified H circle of Leishmania. EMBO J 11: 3601–3608. 1396560 10.1002/j.1460-2075.1992.tb05444.x PMC556819

[68] El Fadili A, Kundig C, Ouellette M (2002) Characterization of the folylpolyglutamate synthetase gene and polyglutamylation of folates in the protozoan parasite Leishmania. Mol Biochem Parasitol 124: 63–71. 12387851 10.1016/s0166-6851(02)00163-9

[69] Sambrook J, Fritsch EF, Maniatis T (1989) Molecular cloning: Cold spring harbor laboratory press New York.

[70] Dumas C, Ouellette M, Tovar J, Cunningham ML, Fairlamb AH, et al (1997) Disruption of the trypanothione reductase gene of Leishmania decreases its ability to survive oxidative stress in macrophages. EMBO J 16: 2590–2598. 9184206 10.1093/emboj/16.10.2590 PMC1169870

