# Supplementary figures and images for: Formation of Linear Amplicons with Inverted Duplications in Leishmania Requires the MRE11 Nuclease
Source: PLoS Genet. 2014 Dec 4;10(12):e1004805. doi: 10.1371/journal.pgen.1004805 (PMC4256157; doi:10.1371/journal.pgen.1004805)

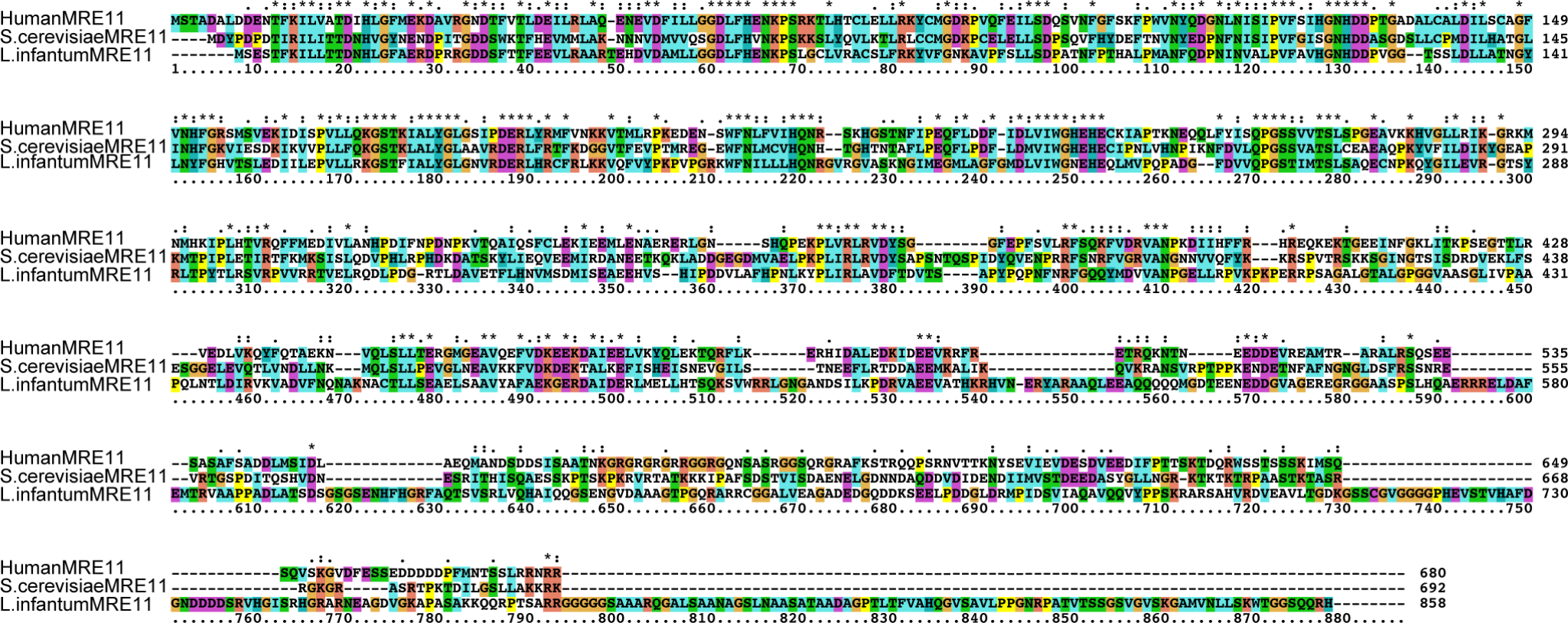

Supplement: Figure S1 — Alignment with ClustalX of MRE11 protein sequences from human, Saccharomyces cerevisiae and Leishmania infantum. Blue represents residues: ACFIMVW; dark blue: HY; pink: ED; green: NQST; yellow: P; orange: G; coral: KR; “*” indicates position which have a single fully conserved residue; “:” indicates a strong group of conserved amino acids; “.” indicates a weaker group of conserved amino acids. (TIF) [file pgen.1004805.s001.tif]

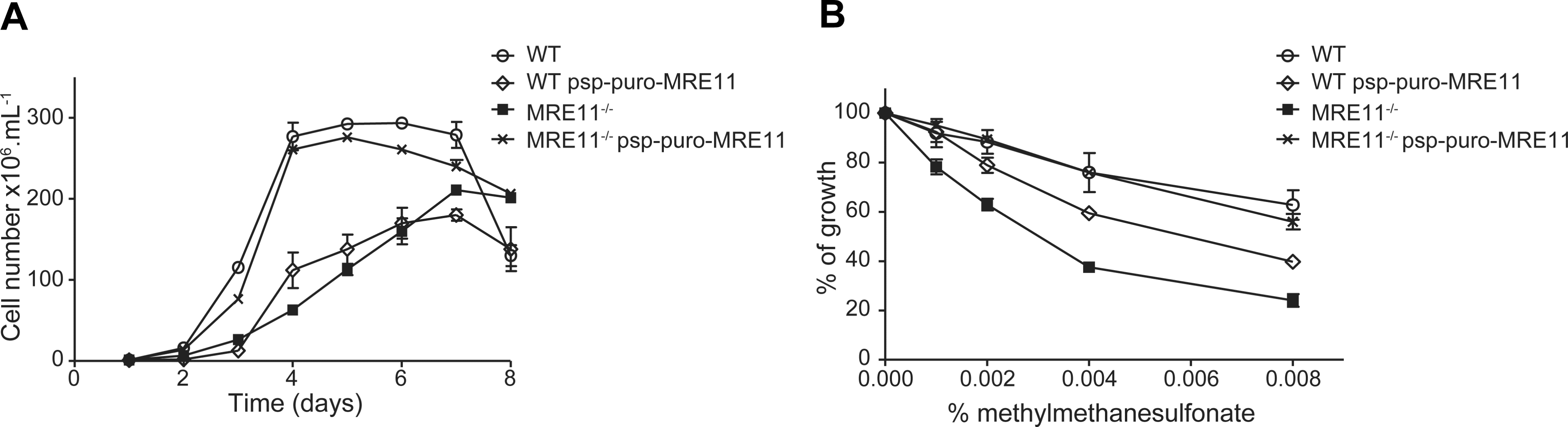

Supplement: Figure S2 — Episomal expression of MRE11 in WT cells and in MRE11−/− L. infantum. Overexpression of LiMRE11WT derived from an episomal construct in L. infantum WT is impairing cell growth (A) and increases sensitivity to MMS (B). The episomal expression of MRE11, however rescued the growth (A) and sensitivity phenotypes (B) of the MRE11−/− parasites. Leishmania infantum WT strain (white circles); L. infantum HYG/NEO MRE11−/− (black squares); L. infantum WT in which an episomal expressing LiMRE11WT construct has been transfected (white diamonds); strain HYG/NEO MRE11−/− in which an episomal expressing LiMRE11WT construct has been transfected (black X). (TIF) [file pgen.1004805.s002.tif]

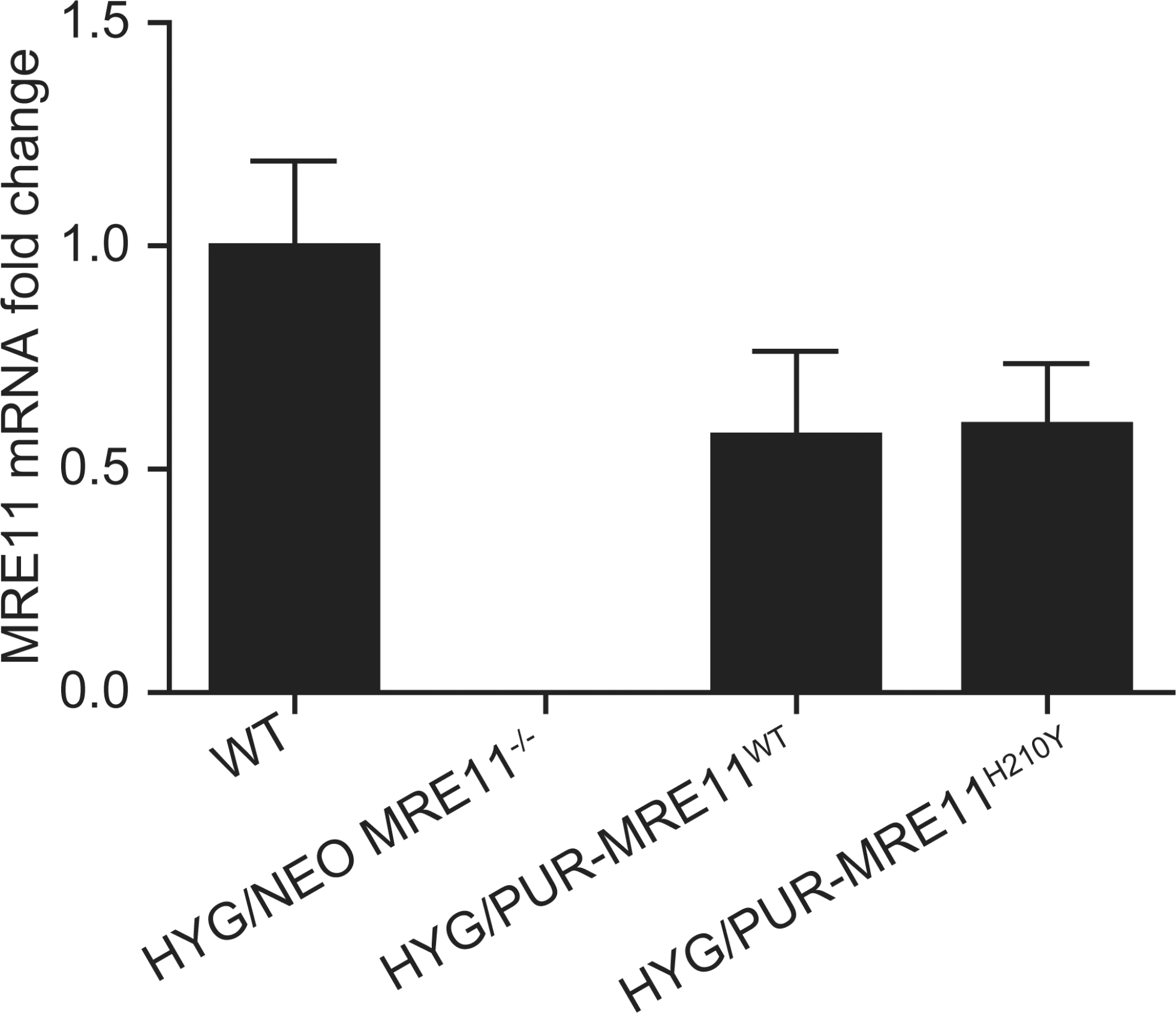

Supplement: Figure S3 — MRE11 RNA expression in Leishmania cells. MRE11 mRNA levels were analyzed by quantitative real-time RT-PCR. The MRE11 RNA expression ratios were normalized to GAPDH expression. (TIF) [file pgen.1004805.s003.tif]

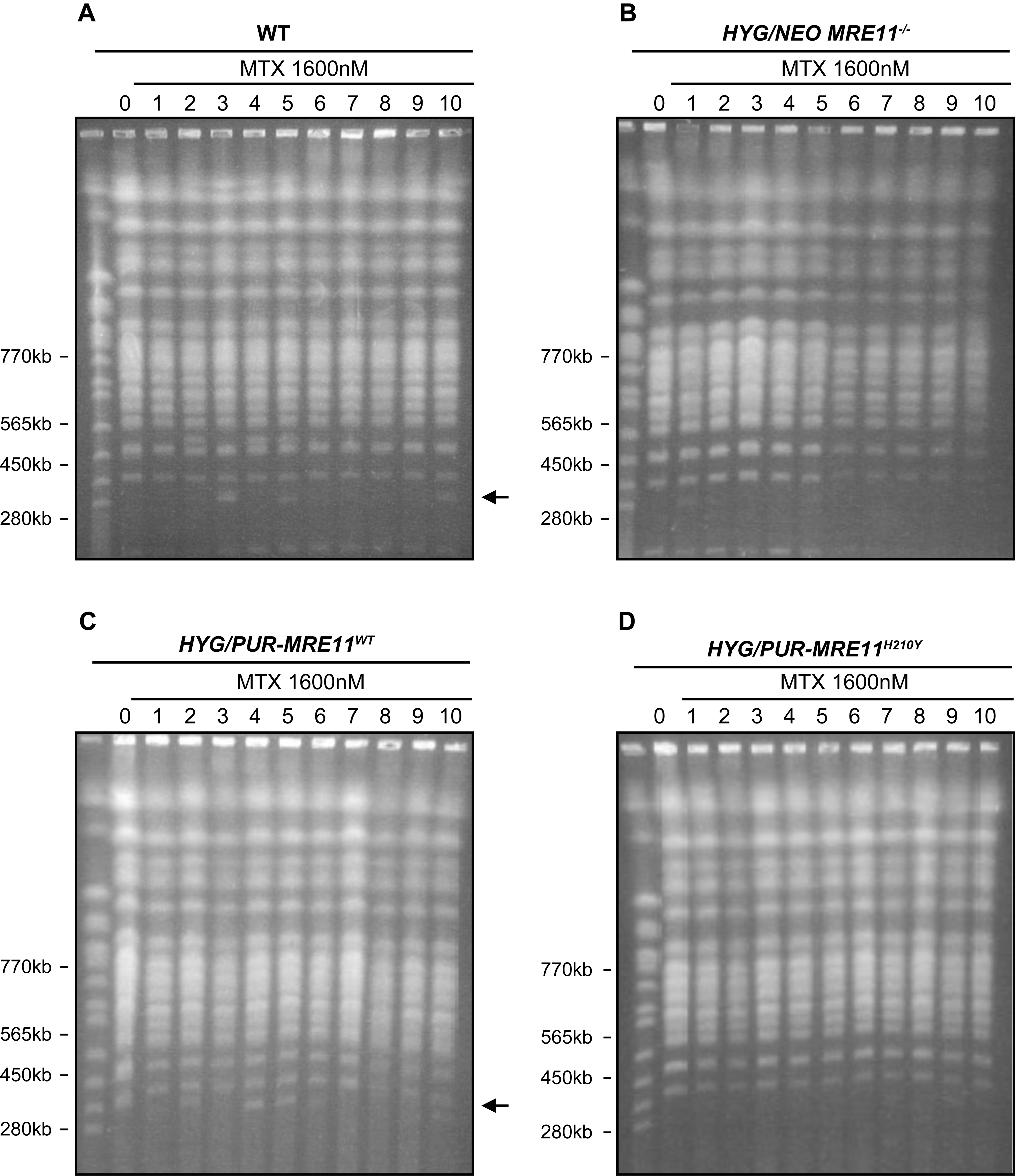

Supplement: Figure S4 — MTX-resistant clones derived from the WT and the HYG/PUR-MRE11 WT strains display DNA bands smaller than the smallest genomic chromosome (indicated by arrows) that correspond to linear amplicons of 300 kb. L. infantum chromosomes were separated by pulsed-field gel electrophoresis using a separation range between 150 kb and 1500 kb and incubated with ethidium bromide. MTX-resistant clones resistant to 1600 nM MTX derived from the WT (A), the HYG/NEO MRE11−/− cells (B), the HYG/PUR-MRE11 WT cells (C) and the HYG/PUR-MRE11 H210Y cells (D). Lanes 0 are parasites without drug selection. (TIF) [file pgen.1004805.s004.tif]

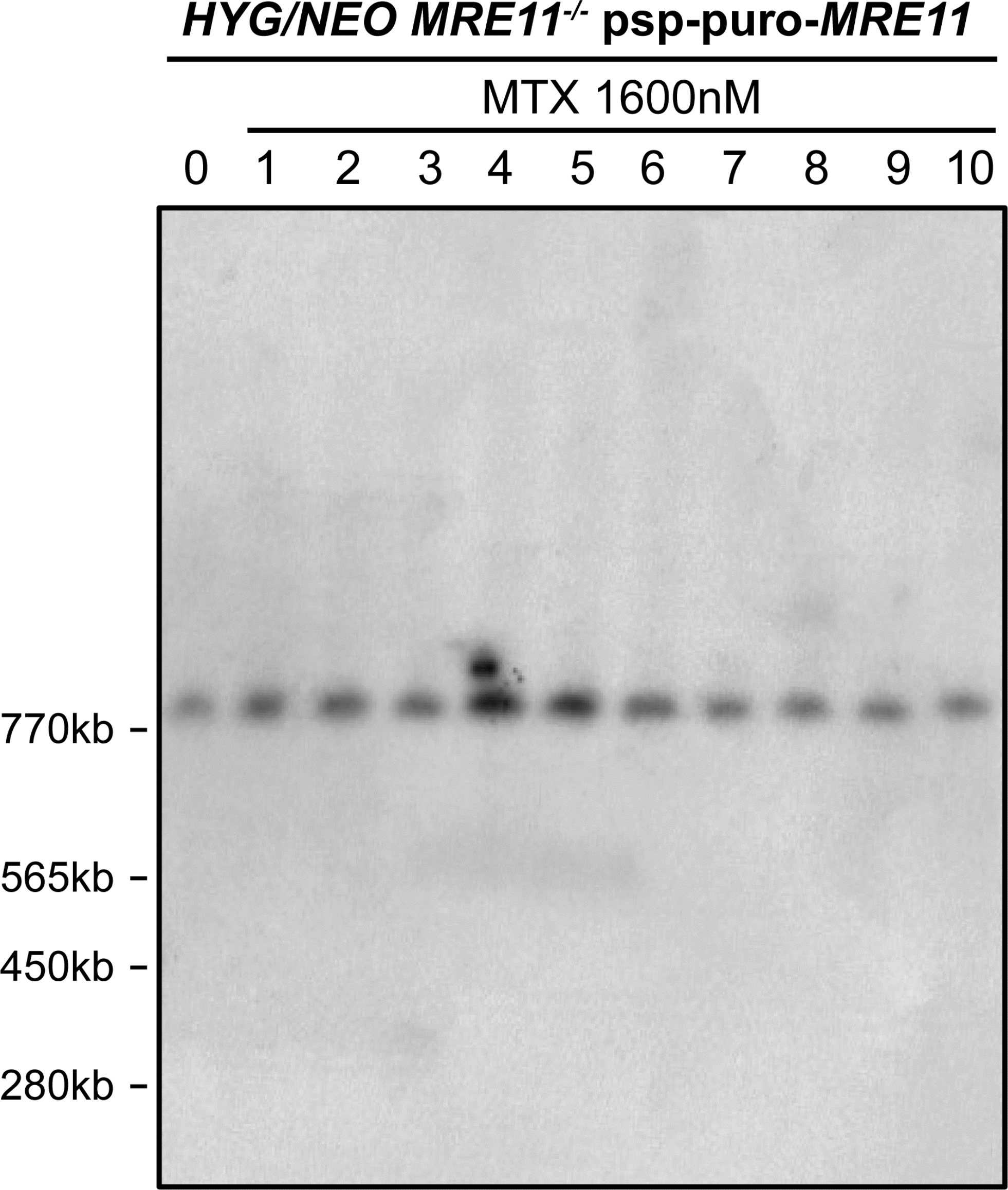

Supplement: Figure S5 — Lack of PTR1 gene amplification in L. infantum MRE11−/− cells complemented with an episomal MRE11 selected for methotrexate (MTX) resistance. HYG/NEO MRE11−/− Psp72-α-puro-α-MRE11 cells were selected for MTX resistance, and their chromosomes were separated by pulsed-field gel electrophoresis using a separation range between 150 kb and 1500 kb. The blot was transferred on membranes and hybridized with a PTR1 probe. Lanes 0 are parasites without drug selection. (TIF) [file pgen.1004805.s005.tif]

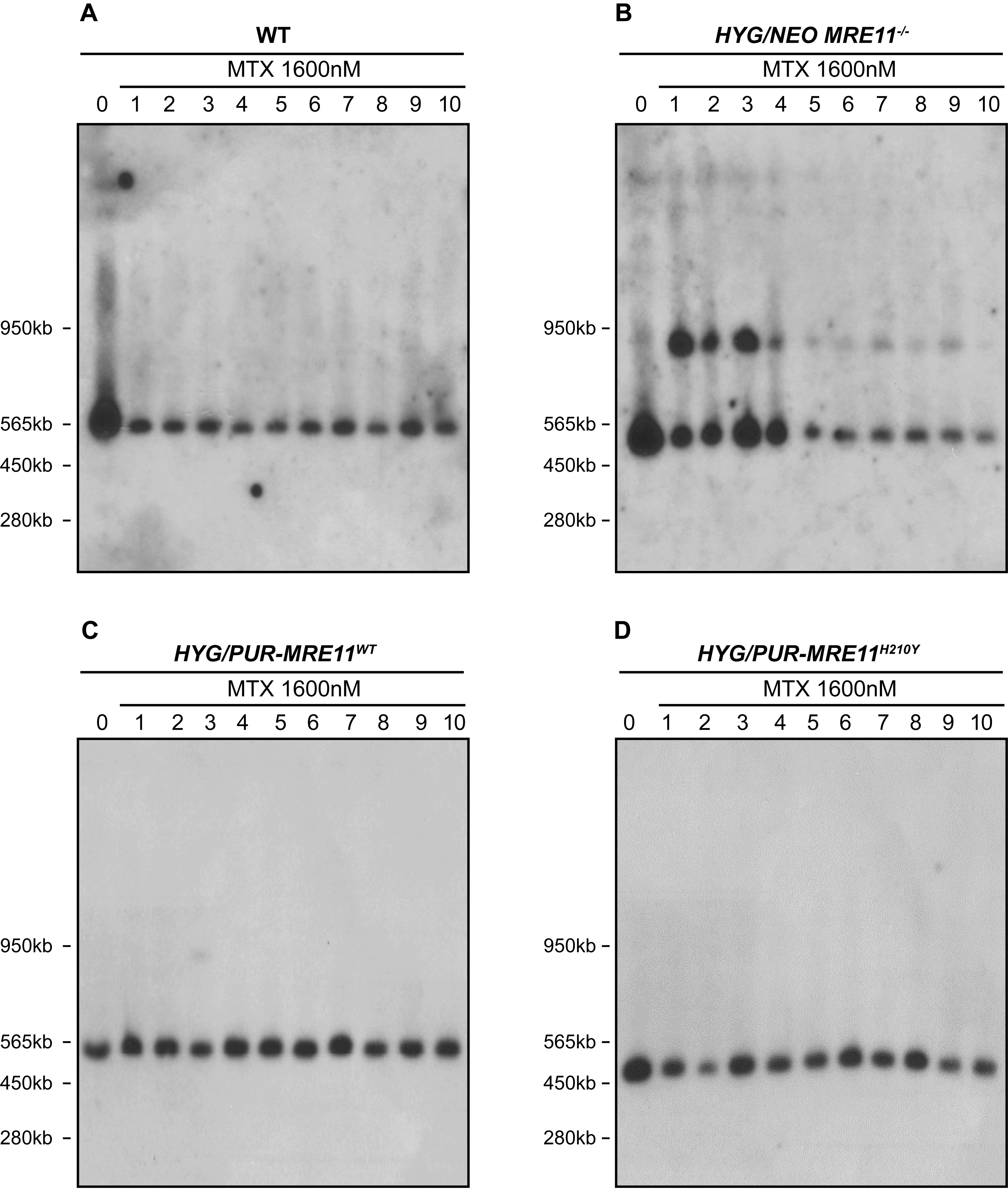

Supplement: Figure S6 — DHFR-TS gene rearrangement of L. infantum MRE11−/− cells selected for methotrexate (MTX) resistance. L. infantum cells were selected for MTX resistance, and their chromosomes were separated by pulsed-field gel electrophoresis using a separation range between 150 kb and 1500 kb, transferred on membranes then hybridized with a DHFR-TS probe. MTX-resistant clones resistant to 1600 nM MTX derived from the WT (A), the HYG/NEO MRE11−/− cells (B), the HYG/PUR-MRE11 WT cells (C) and the HYG/PUR-MRE11 H210Y cells (D). Lanes 0 are parasites without drug selection. (TIF) [file pgen.1004805.s006.tif]

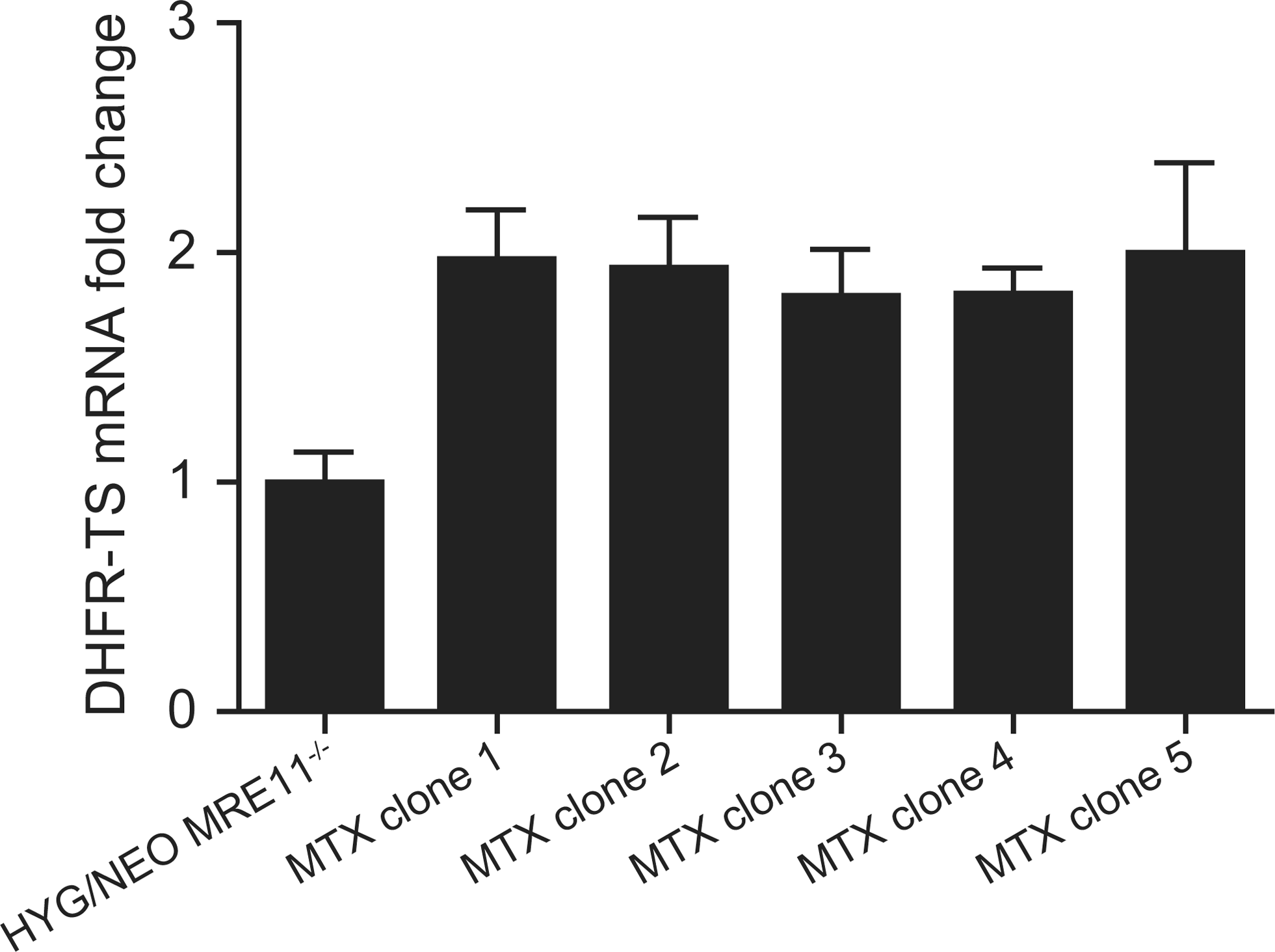

Supplement: Figure S7 — DHFR-TS RNA expression in MRE11−/− cells selected for MTX resistance. The RNAs derived from the MRE11−/− and from five MRE11−/− methotrexate resistant clones were analyzed by quantitative real-time RT-PCR. The DHFR-TS RNA expression ratios were normalized to GAPDH expression. (TIF) [file pgen.1004805.s007.tif]
