# Supplementary material for: Formation of Linear Amplicons with Inverted Duplications in Leishmania Requires the MRE11 Nuclease
Source: PLoS Genet. 2014 Dec 4;10(12):e1004805. doi: 10.1371/journal.pgen.1004805 (PMC4256157; doi:10.1371/journal.pgen.1004805)
Supplement: Table S1 — Primers used in this study were designed using PrimerQuest software. (DOCX) [file pgen.1004805.s008.docx]

**Table S1.** Primers used in this study.

| **Primer name** | **Sequence (5'-3')** |
| --- | --- |
| 1 | ATGTCTGAGAGCACCTTCAA |
| 2 | CTAGTGCCGCTGCTGAGATC |
| 3 | CGGCCAACGAACCTCTGGCA |
| 4 | AATCCATCTTGTTCAATCATCCTGTGAGCAAGAGCGGACG |
| 5 | GTGAGTTCAGGCTTTTTCATCCTGTGAGCAAGAGCGGACG |
| 6 | ACGGCGGCAGCAGCGGCTCCCTAGTGCCGCTGCTGAGATC |
| 7 | ATGATTGAACAAGATGGATTGCACGC |
| 8 | ATGAAAAAGCCTGAACTCACCGCGA |
| 9 | CTGTCCAAGTGGACAGGTGGATCTCAGCAGCGGCACTAGG |
| 10 | CCACACGGCCGCCGCTCGTGTCAGAAGAACTCGTCAAGAAG |
| 11 | CCACACGGCCGCCGCTCGTGTCATCGATGATGGGGATCTG |
| 12 | CCACACGGCCGCCGCTCGTGTCAGGCACCGGGCTTGCGGG |
| 13 | CACGAGCGGCGGCCGTGTGGTTGC |
| 14 | GCAGCGGGAGGAGGGGGAGG |
| 15 | CCGCAAGTGGTTTAATATCCTGCTACTGTATCAGAACCGCGGCGTGCGTGGCGTGGCGA |
| 16 | TCGCCACGCCACGCACGCCGCGGTTCTGATACAGTAGCAGGATATTAAACCACTTGCGG |
| 17 | ACGGCATAAAGCTTGACGATTACATTGCTAGGACATCTTTGCCCACCTGCAGGTTCACCC |
| 18 | CATAAAGCTTGACGATTACATTGCTAGGACATCTTTGCCCACCTGCAGGTTCACCC |
| 19 | ATCTACGGCATAAAGCTTGACGATTACATTGCTAGGACATCTTTGCCCACCTGCAGGTTCACCC |
| 20 | ACGCTGCCGAATTCTACCAGTGCCAGCGACGGACATCTTTGCCCACCTGCAGGTTCACCC |
| 21 | GGGTGAACCTGCAGGTGGGCAAAGATGTCCTAGCAATGTAATCGTCAAGCTTTATGCCGT |
| 22 | TCTTGGTTTAACTTATTTGTGATTTATCAGAACAGGAGTAAACATGGAAGT |
| 23 | ACTTCCATGTTTACTCCTGTTCTGATAAATCACAAATAAGTTAAACCAAGA |
| a | ACCCTTTGCCTGTACACGTGCC |
| a’ | CTAGTCCGTGTGCTGGCTTGCG |
| b | CGCGTGCGTCTGAGTGAGAACC |
| b’ | CGGCTGTCACCACCTCATCTGC |
| c | GAGGGAGTGCACGGACGGGAG |
| c’ | CGGTGCCACGTCGGTCAAAACA |
| d | GAGGGAGTGCACGGACGGGAG |
| d’ | CGGCTGTCACCACCTCATCTGC |
| e | CGGTGCCACGTCGGTCAAAACA |
| e’ | CGCGTGCGTCTGAGTGAGAACC |
| f | TCAGCGTGTTGCTACGGTCCAGAG |
| f’ | TGTCTAGGGCTACTTCGGCACCAC |
| g | GACGGGGCACTTGTTGTGAA |
| g’ | CAGCGAGATGCCGGCTTTGG |
| GAPDH qRT-PCR forward | GTACACGGTGGAGGCTGTG |
| GAPDH qRT-PCR reverse | CCCTTGATGTGGCCCTCGG |
| MRE11 qRT-PCR forward | ACCTCAACTACTTTGGCCACGTCA |
| MRE11 qRT-PCR reverse | AGGCACTGGCTTCGGATAGACAAA |
| DHFR-TS qRT-PCR forward | GGGTTTGAAGCGAACTACGA |
| DHFR-TS qRT-PCR reverse | GGGTTCCAGGCAGTGAATAG |
